# Straight motion of half-integer topological defects in thin Fe-N magnetic films with stripe domains

**DOI:** 10.1038/s41598-018-27283-7

**Published:** 2018-06-19

**Authors:** S. Fin, R. Silvani, S. Tacchi, M. Marangolo, L.-C. Garnier, M. Eddrief, C. Hepburn, F. Fortuna, A. Rettori, M. G. Pini, D. Bisero

**Affiliations:** 1 Dipartimento di Fisica e Scienze della Terra, Università degli Studi di Ferrara, Via Saragat 1, I-44122 Ferrara, Italy; 2 Dipartimento di Fisica e Geologia, Università degli Studi di Perugia, Via Pascoli, I-06123 Perugia, Italy; 3 Istituto Officina dei Materiali del CNR (CNR-IOM), Sede Secondaria di Perugia, c/o Dipartimento di Fisica e Geologia, Università degli Studi di Perugia, I-06123 Perugia, Italy; 4 Sorbonne Université, CNRS, Institut des NanoSciences de Paris, UMR 7588, F-75252 Paris, France; 5 Université Versailles St-Quentin, LISV, Bâtiment Boucher, Pôle scientifique et technologique de Vélizy, 10-12 avenue de l’Europe, F-78140 Vélizy, France; 6 CSNSM, Université Paris-Sud and CNRS/IN2P3, Université Paris-Saclay, F-91405 Orsay, France; 7 Dipartimento di Fisica ed Astronomia, Università degli Studi di Firenze, Via Sansone 1, I-50019 Sesto Fiorentino, FI Italy; 8 Istituto dei Sistemi Complessi del CNR (CNR-ISC), Sede Secondaria di Sesto Fiorentino, Via Madonna del Piano 10, I-50019 Sesto Fiorentino, FI Italy; 9 CNISM, Unità di Ferrara, I-44122 Ferrara, Italy

## Abstract

In thin magnetic films with perpendicular magnetic anisotropy, a periodic “up-down” stripe-domain structure can be originated at remanence, on a mesoscopic scale (~100 nm) comparable with film thickness, by the competition between short-range exchange coupling and long-range dipolar interaction. However, translational order is perturbed because magnetic edge dislocations are spontaneously nucleated. Such topological defects play an important role in magnetic films since they promote the in-plane magnetization reversal of stripes and, in superconductor/ferromagnet hybrids, the creation of superconducting vortex clusters. Combining magnetic force microscopy experiments and micromagnetic simulations, we investigated the motion of two classes of magnetic edge dislocations, randomly distributed in an ${{\rm{N}}}_{2}^{+}$-implanted Fe film. They were found to move in opposite directions along straight trajectories parallel to the stripes axis, when driven by a moderate dc magnetic field. Using the approximate Thiele equation, analytical expressions for the forces acting on such magnetic defects and a microscopic explanation for the direction of their motion could be obtained. Straight trajectories are related to the presence of a periodic stripe domain pattern, which imposes the gyrotropic force to vanish even if a nonzero, half-integer topological charge is carried by the defects in some layers across the film thickness.

## Introduction

The formation of domain patterns^1^ in the stripe morphology has been observed in a variety of physical systems, such as high-T_*c*_ superconductors^2^, mixtures of lipids in monolayers at the air-water interface^3^, self-assemblies of lamellar diblock copolymers^4^, and two-dimensional electron systems^5^. In all these cases, spontaneous phase separation on a mesoscopic scale takes place as the result of competing interactions acting on different spatial scales^1,6–8^. In such a context, ferromagnetic thin films represent a paradigmatic case where a periodic domain structure may be stabilized by the competition between short-range exchange coupling, favouring parallel alignment between two spins, and long-range magnetic dipole-dipole interaction, favouring antiparallel alignment. In the presence of a perpendicular magnetic anisotropy (PMA), the domains have opposite out-of-plane (z) magnetization components. In zero applied field, these “up” and “down” domains are self-organized into stripes of equal width, and the net z-magnetization vanishes. The amount of PMA is measured by the quality factor, *Q*, defined as the ratio between the uniaxial out-of-plane anisotropy (*K*_*u*_) and the shape anisotropy (${{K}}_{{d}}=2\pi {{M}}_{{s}}^{2}$, where *M*_*s*_ is the saturation magnetization). Ferromagnetic films with *Q* >> 1 can develop stripe domains for extremely low thickness ($t\lesssim 1$ nm)^9–13^. Whereas, in films with a moderate PMA (*Q* < 1), stripe domains can form only for *t* > *t*_*c*_, where the critical thickness, *t*_*c*_, is proportional^14^ to the Bloch wall width of the material (${{l}}_{{B}}=\sqrt{{{A}}_{{\rm{ex}}}/{{K}}_{{u}}}$, where *A*_ex_ is the exchange energy per unit length). Experimental values of *t*_*c*_ typically range from 20 to 200 nm^14–16^ and the period of the stripe pattern, *P*, is found^15,16^ to increase as $\sqrt{t}$. Moreover, in ferromagnetic films with PMA, a clear evidence for topological defects in the stripe domain structure was found. In ultrathin^9,12^ and thin^17^ samples grown with epitaxial accuracy on top of a non magnetic single-crystal surface, such defects were observed using different imaging techniques, such as magnetic force microscopy^17^, scanning electron microscopy with polarization analysis^9^, and magnetic circular dichroism^12^. Recently, quantitative x-ray magnetic microscopy has been exploited to obtain nanoscale imaging of buried topological defects^18^ in thin films^18^ and multilayers^19,20^ grown by dc magnetron sputtering. Using this method, the defects were identified as complex objects carrying a half-integer topological charge (merons)^18,19^. Further x-ray microscopy data in magnetic trilayers, supported by micromagnetic simulations, showed that dislocations in the stripe pattern drive the nucleation of reversed magnetic domains under the effect of pulsed magnetic fields^20^. Moreover, in superconductor/ferromagnet thin film heterostructures^21^, these defects (i.e., magnetic edge dislocations) have recently been found to produce an enhancement of the out-of-plane magnetization in the region where a magnetic stripe bifurcates, with the effect of promoting the local accumulation of superconducting vortices.

In this work, we study the motion of topological defects that develop, in the form of magnetic edge dislocations, in the stripe domain structure of nitrogen-implanted iron (Fe-N) thin films with a moderate PMA^22^. By epitaxially growing Fe on a single crystal of ZnSe/GaAs(001), and using an opportune dose of ${{\rm{N}}}_{2}^{+}$ ions for implantation^23^, it is possible to obtain straight and well-defined magnetic stripe domains with a few isolated edge dislocations. It is worth remarking that, in our Fe-N films, no experimental evidence was found for the inversion-symmetry-breaking Dzyaloshinskii-Moriya^24,25^ interaction (DMI), which is known to be an important factor for the emergence and stabilization of topological defects in many spin systems^26–28^. Interestingly, dipole-stabilized skyrmions and skyrmion lattices have recently been engineered^29,30^ in Fe/Gd films and multilayers with PMA but without DMI, upon application of a perpendicular magnetic field. In contrast, in our Fe-N films, the topological defects, in the form of magnetic edge dislocations of the periodic stripe domain pattern, are found to nucleate spontaneously, at room temperature, as remanence is approached^23^. The breaking of long-range order from topological defects in the stripe domain phase can be attributed^9^ both to “quenched” disorder (associated with ever-present atomic imperfections at the Fe-N film surface) and “self-generated” disorder (intrinsic to the stripe formation process, in systems with competing interactions acting on different spatial scales)^6–8,31,32^. In particular, combining magnetic force microscopy (MFM) measurements and micromagnetic simulations, we study the driving effect exerted on magnetic edge dislocations by an external magnetic field. Two classes of magnetic dislocations are observed, moving straight in opposite directions, when a static magnetic field of moderate intensity is applied in plane along the stripe axis. Using an approximate theoretical approach based on the Thiele equation, we are able to analytically calculate the forces acting on the defects, and give a microscopic explanation for the direction of their motion. The straight trajectory of a magnetic edge dislocation is due only to the external force and the dissipative force, which are both parallel to the stripes axis. Whereas, the gyrotropic force is shown to vanish owing to the presence of the periodic stripe domain pattern even if a nonzero, half-integer topological charge is carried by the dislocation in some layers across the film thickness.

## Results

### MFM experiments in Fe-N films with stripe domains

**Figure 1:**
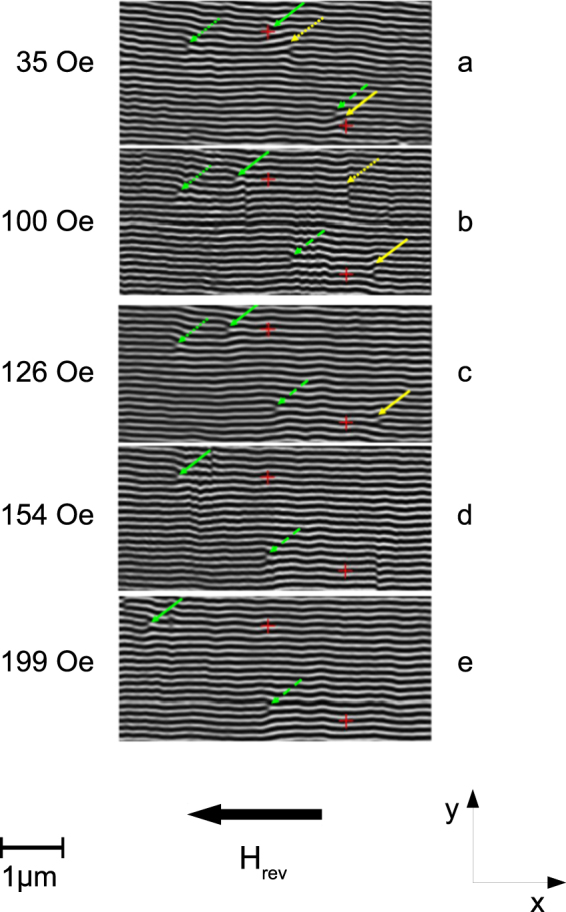
MFM experimental investigation of magnetic edge dislocations motion in the Fe-N film. The MFM images show the evolution of the domain pattern at the surface of a 78 nm thick Fe-N film in the presence of a reversal field, **H**_rev_, applied along the stripes axis. Red crosses mark fixed positions on the surface of the sample, which are a reference for the determination of the motion of magnetic dislocations (see Supplementary Information). On increasing the field intensity, two different types of magnetic edge dislocations (green and yellow arrows) are found to translate into opposite directions along the stripes axis (i.e., parallel and antiparallel to **H**_rev_, respectively). The bar is the unit length of 1 *μ*m.

An Fe-N film, 78 nm thick, was prepared on a GaAs(001) substrate following the procedure described in Methods, based on N-implantation of an MBE-grown Fe thin film. The film thickness is higher than the critical value, *t*_*c*_, which has to be exceeded for the formation of magnetic stripe domains. For Fe-N films, an approximate analytical estimate^14^ provides ${t}_{c}=2\pi \sqrt{{A}_{{\rm{e}}x}/{K}_{u}}\sim 42$ nm, where^22,23^ ${A}_{ex}=2.16\times {10}^{-6}$ erg/cm is the exchange constant and ${K}_{u}=4.9\times {10}^{6}$ erg/cm^3^ is the perpendicular magnetic anisotropy constant. The saturation magnetization is ${M}_{s}=1700$ emu/cm^3^, and the quality factor is $Q={K}_{u}/{K}_{d}={K}_{u}\mathrm{/2}\pi {M}_{s}^{2}=0.27 < 1$. In order to perform MFM measurements, the sample was initially saturated in plane, applying a strong magnetic field (*H*_sat_ = 4 kOe) along the [−1, 1, 0] crystallographic direction. Then, the field was removed, so as to nucleate the stripe structure along that direction, and an increasing magnetic field **H**_rev_ was applied in-plane in the direction antiparallel to the saturation field, to study the displacement of magnetic edge dislocations. Figure 1 shows MFM images taken in the presence of the reversal field **H**_rev_. Red crosses mark fixed positions on the surface of the sample, covered with an 8 nm thick Au layer, taken exploiting the imperfections of the topography as deduced from atomic force microscopy (AFM) images. In fact, using AFM it was possible to identify a characteristic group (“archipelago”) of contaminant particles that adhere to the surface of the Au capping layer maintaining fixed positions between one scan and another. Placing the origin of a Cartesian coordinate system on one of these particles, it was possible to determine the coordinates of each point of the examined area, which are obviously common to both the AFM image and the MFM one. This allowed to mark any magnetic configuration at a certain field and follow its displacement at different fields. A schematic example of the method used to measure the position of magnetic edge dislocations, based on the simplest possible archipelago (two particles), is described in the Supplementary Information (Section I). However, it is important to note that the velocity of the dislocations could not be measured, because each MFM image requires ~8 min to be completed. From the fast Fourier transform of the images, the period of the stripe pattern was found to be *P* = 101 nm. The analysis of the field-driven motion of magnetic edge dislocations is somewhat complicated by the presence of other types of structures which perturb the periodicity of the stripe pattern, such as ripples and phase jumps. These structures, which are visible respectively on the top right corner of Fig. 1b and on the bottom right corner of Fig. 1d, might be attributed to a spontaneous (i.e., not driven by external stimuli) relaxation mechanism, occurring in the system on a timescale which cannot be captured by MFM. Nevertheless, when comparing all the images in Fig. 1(a–e), a general tendency can be perceived as interpretable in the frame of field-driven effects. Namely, on increasing the intensity, **H**_rev_, of the reversal magnetic field (yet smaller than the coercive field, *H*_*c*_), two classes of magnetic edge dislocations, marked by green and yellow arrows respectively, appear to be displaced along the stripes axis in opposite directions. The magnetic edge dislocations marked by the green arrows clearly appear to be driven along the same direction as the external applied field **H**_rev_ (i.e. towards the left side of the MFM image). Note that the dislocations marked by the green full and long-dashed arrows can be followed in their displacements for all the investigated field values, while the dislocation marked by the green short-dashed arrow moves out of the image for ${H}_{{\rm{rev}}}\ge 154$ Oe. The magnetic edge dislocations marked by the yellow arrows, instead, are driven along the direction antiparallel to **H**_rev_ (i.e. towards the right side of the MFM image). The dislocations marked by the yellow full and short-dashed arrows can be followed up to **H**_rev_ = 126 and 100 Oe, respectively; for larger fields, they are found to move out of the MFM images. Moreover, it appears that the dislocations may be displaced by different amounts for the same increment of the in-plane field intensity. This behaviour can be related to the presence of structural defects, which in a real Fe-N film occupy fixed positions (between one MFM scan and another) randomly distributed throughout the sample. Such structural defects are expected to behave as pinning centers for the dc magnetic field-driven motion of magnetic edge dislocations, similarly to the case recently investigated^33,34^ of current-driven magnetic skyrmions in ultrathin metallic ferromagnets. On the one hand, pinning centers are likely to arrest the field-driven motion of magnetic edge dislocations in a random way; on the other hand, a random distribution is expected, for the intensity of the depinning fields of magnetic edge dislocations. Consequently, the field-driven displacements of magnetic edge dislocations in the real Fe-N film cannot be uniform. Finally, it is worth mentioning that further MFM data were obtained in the same Fe-N sample at remanence (i.e., after removal of a dc magnetic field, applied with different signs and intensities). Interestingly, the data showed that some magnetic edge dislocations exist, for which the inversion of the in-plane field induces an inversion in their displacement along the stripes axis (see Supplementary Information, Section II).

### Micromagnetic simulations of the motion of magnetic edge dislocations

**Figure 2:**
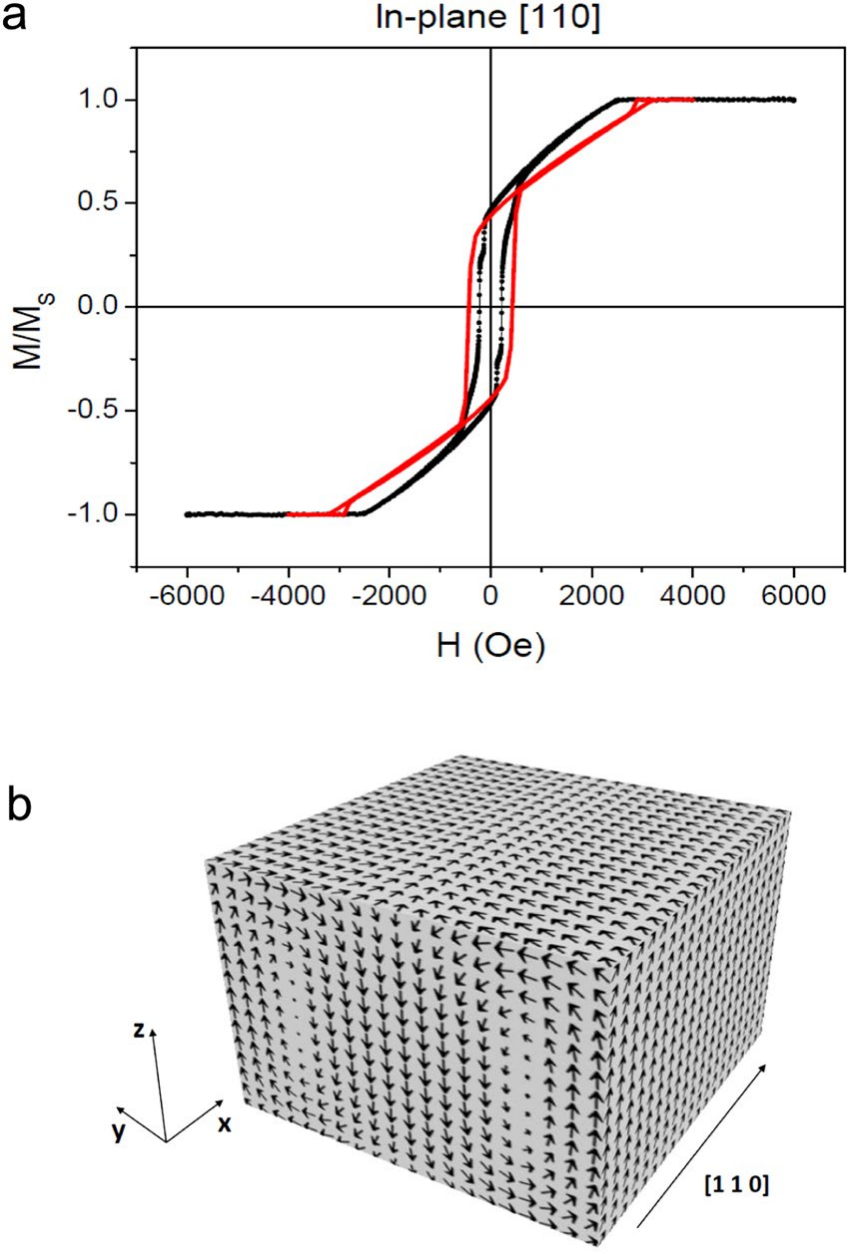
Hysteresis loop and magnetization distribution in the Fe-N film at remanence. (**a**) Magnetization loop of the Fe-N film, as measured by VSM (black dots) for magnetic field applied in plane along [110], and as calculated by micromagnetic simulations (red line). (**b**) Simulated magnetization distribution at remanence, reducing to zero the magnetic field applied in the film plane, *xy*, along the stripes axis, *x*.

**Figure 3:**
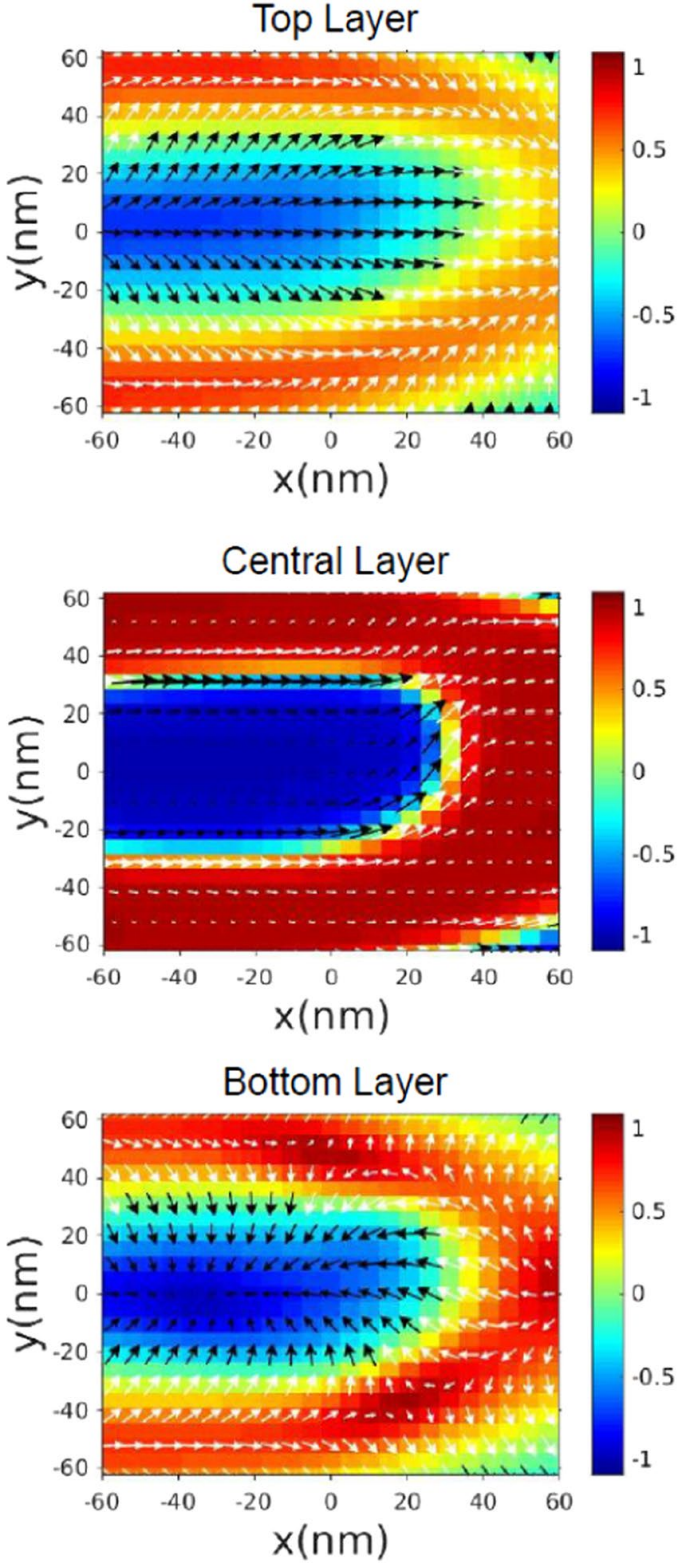
Simulated magnetization distribution of a magnetic edge dislocation across film thickness. The simulated magnetization distribution of a magnetic edge dislocation is shown in the top, central and bottom layer of the film, respectively. Black/white arrows denote the in-plane unit magnetization vector **n**_IP_ = (*n*_*x*_, *n*_*y*_), while the colours in the vertical scale (r.h.s. of each panel) provide *n*_*z*_. The simulated dislocation displays a nearly semicircular shape, with the diameter approximately equal to the width of a stripe (≈50 nm).

To obtain a deeper insight into the displacement of magnetic edge dislocations, micromagnetic simulations were carried out by numerically solving the Landau-Lifshitz-Gilbert (LLG) equations of motion^35^. The simulated in-plane hysteresis loop (red line) is reported in Fig. 2a, showing a nice agreement with vibrating sample magnetometry (VSM) measurements (black dots) performed for magnetic field ${\bf{H}}\Vert \mathrm{[110]}$. When *H* is reduced from positive saturation, a linear region (indicating the formation of stripe domains^17^ along the [110] axis) is numerically found starting at $H\approx 3$ kOe. Also the simulated remanent magnetization $({M}_{r}/{M}_{s}\approx 0.5)$ and coercive field (${H}_{c}\approx 400$ Oe) are found to compare rather well with experiment. Let us denote the unit magnetization vector **M**/*M*_*s*_ by ${\bf{n}}=({n}_{x},{n}_{y},{n}_{z})$. The magnetization distribution, calculated at positive remanence, is displayed in Fig. 2b in a region far from a magnetic edge dislocation. One finds that, between two domains with opposite out-of-plane magnetization components (*n*_*z*_ > 0 and *n*_*z*_ < 0), in the central region of the film there are transition regions (Bloch walls) with in-plane magnetization always directed along the stripes axis (*n*_*x*_ > 0); whereas, near the two film surfaces there are flux-closure magnetic domains (Néel caps) with opposite in-plane magnetization components (*n*_*y*_ > 0 and *n*_*y*_ < 0). In good agreement with the experimental results, the calculated period of the stripes is *P* = 100 nm, and it remains constant on changing the intensity of the applied field. In the micromagnetic simulations, the topological defects in the stripe domain pattern are generated in the form of a pair of magnetic edge dislocations. Figure 3 shows the simulated magnetization distribution on the top surface layer, the central layer and the bottom surface layer for one of the dislocations in the pair. Black/white arrows denote the in-plane magnetization **n**_IP_ = (*n*_*x*_, *n*_*y*_), while the colours (see the vertical scale on the r.h.s. of each panel) provide *n*_*z*_. The simulated dislocation displays a nearly semicircular shape, with the diameter approximately equal to the width of a stripe ($2R=P\mathrm{/2}\approx 50$ nm). In the top surface layer the in-plane magnetization is directed outwards starting from the center of the semicircle, while in the bottom surface layer it is directed inwards. Such a peculiar magnetization distribution can be explained observing that closure domains (Néel caps) with in-plane orientation of the magnetization are located near the two film surfaces, in the transition region between two domains with opposite z-polarization. Approaching the central film layers, instead, n_IP_ tends to become nearly tangential to the semicircumference because Néel caps at the film surfaces are replaced by Bloch walls in the central region of the film. A similar magnetization distribution has been observed for the other magnetic edge dislocation in the pair.

**Figure 4:**
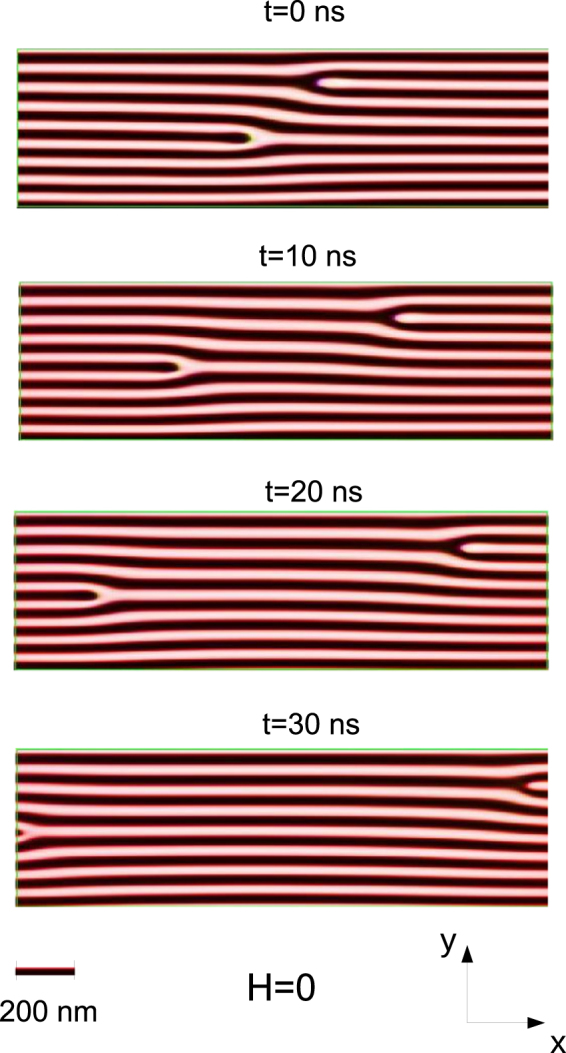
Simulated motion of a pair of magnetic edge dislocations along the stripes axis. Time equispaced (10 ns) snapshots, showing the motion of a pair of dislocations along straight trajectories at remanence. The two dislocations move into opposite directions and eventually exit from the sample for *t* > 35 ns, leaving a perfectly ordered magnetic stripe domain structure. Here white/black colours denote regions with up/down out-of-plane magnetization. Whereas, in the red region, the magnetization is in plane. The bar is the unit length of 200 nm.

**Figure 5:**
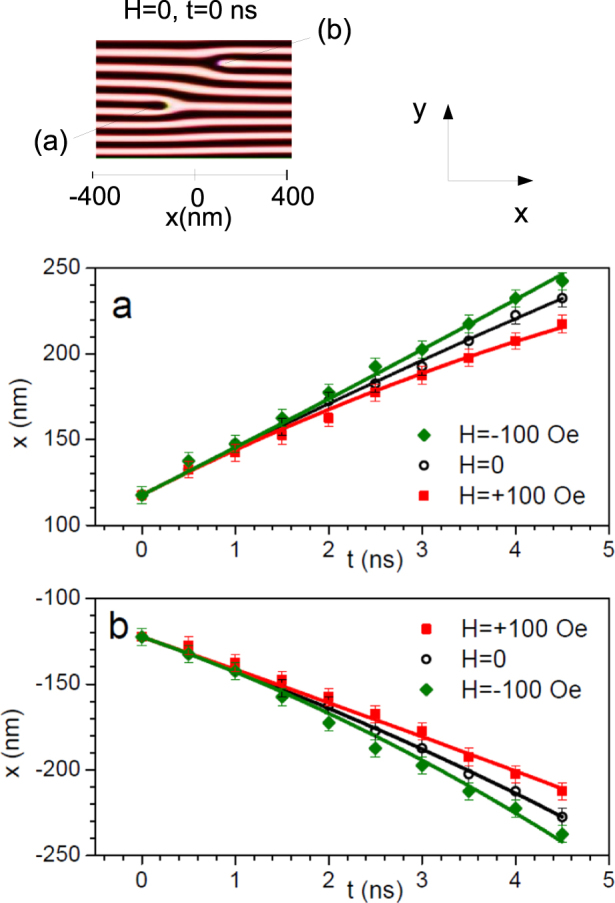
Kinematic equations for the motion of a pair of dislocations along the stripes axis. Top: Initial stripe domain configuration at remanence, showing a pair of opposite magnetic edge dislocations, (a) and (b). Bottom: Fittings, for *H* = 0 and ±100 Oe, of the simulated motion of each dislocation in a time interval of 5 ns, where the contribution of internal forces can be assumed to be constant. The straight trajectories were fitted using a kinematic equation with a field-dependent uniform acceleration, *a*_*H*_. For each dislocation, nearly opposite values of the incremental acceleration, Δ*a*_*H*_ = *a*_*H*_ − *a*_*H*=0_, were found upon field inversion.

Subsequently, the motion of a pair of opposite dislocations was simulated^36^. Figure 4 reports time-equispaced (10 ns) snapshots taken from the simulation at *H* = 0 (see Supplementary Movie). The same rectangular portion of film surface is shown in each frame. It appears that the motion takes place along straight trajectories parallel to the stripes axis, in opposite directions for the two dislocations in the pair. For *t* > 35 ns, the two dislocations exit from the sample leaving a perfectly ordered magnetic stripe domain structure. Concerning the zero-field motion of the magnetic edge dislocations, one has to consider that in the micromagnetic simulations structural defects were not included. Consequently, their role as pinning centers, ever present in a real Fe-N sample and capable to affect, or even arrest, the motion^33,34^ of magnetic dislocations, was not taken into account in the calculations. Even more important is that, in the simulations, magnetic edge dislocations (generated as described in Methods) are non-equilibrium objects: after a sufficiently long time the defects exit from the sample, leaving a perfectly ordered magnetic stripe domain structure (Supplementary Movie). Actually, the forces responsible for the zero-field motion of magnetic edge dislocations (Fig. 4) are the internal forces due to exchange coupling, magnetostatic energy and magnetic anisotropy energy. All these forces were taken into account in the numerical solution of the LLG equations and gave rise to a non-stationary motion, even including dissipative effects in an opportune way^36^. In addition, starting from the magnetic configuration obtained at *H* = 0, we investigated (see Supplementary Movie) the motion of the dislocations when an external magnetic field of low intensity (*H* = ±100 Oe) is applied along **e**_x_. Straight trajectories parallel to the stripes axis were found also in this case, with the two dislocations of the pair moving into opposite directions. To unravel the effect of a nonzero in-plane magnetic field *H* on the dislocations motion, we limited the analysis to a very short time interval (5 ns) where the acceleration due to the internal forces can be assumed to be nearly constant. In Fig. 5a and b, we plot the displacement of a pair of dislocations along the stripes axis as a function of time, *x*(*t*). The symbols denote simulated positions, while the curves represent fits obtained using the kinematic equation $x(t)=\frac{1}{2}{a}_{H}{t}^{2}+{v}_{0}t+{x}_{0}$, where *a*_*H*_ is the field-dependent acceleration; *v*_0_ and *x*_0_ are respectively the initial velocity and position, which were assumed to be independent of *H*. Defining the incremental acceleration of a defect as ${\rm{\Delta }}{a}_{H}={a}_{H}-{a}_{H=0}$, we found (a) ${\rm{\Delta }}{a}_{H=+100{\rm{Oe}}}=+\,1.62$ nm/ns^2^, ${\rm{\Delta }}{a}_{H=-100{\rm{Oe}}}=-\,1.44$ nm/ns^2^, for the black interrupted stripe (Fig. 5a); and (b) $\Delta {a}_{H=+\mathrm{100\ }{\rm{O}}e}=-\,1.67$ nm/ns^2^, ${\rm{\Delta }}{a}_{H=-100{\rm{Oe}}}=+\,1.40$ nm/ns^2^, for the white interrupted one (Fig. 5b). Within standard error (± 0.20 nm/ns^2^) in the fit of the incremental acceleration, the two magnetic edge dislocations in the pair are characterized, for fixed *H*, by nearly opposite values of Δ*a*_*H*_. Moreover, for a given dislocation, we found that the increments Δ*a*_*H*_ assumed nearly opposite values upon field inversion. Note that, from the fits in Fig. 5, the effect of a dc magnetic field of intensity *H* = 100 Oe on the displacement of a given dislocation is ${\rm{\Delta }}x(t) \sim 20$ nm = 0.02 *μ*m after only 5 ns. Therefore, even taking into account that dissipative forces reduce the acceleration with respect to the initial value estimated in Fig. 5, if one could pursue the numerical calculations over the space scale and the time scale (~8 min for each MFM image) of experiments, the simulated field-induced displacement would significantly exceed the experimental displacement which, for a 100 Oe field increment, is on the order of a few *μ*m’s (see Fig. 1). Summing up, just as in the case of the zero-field motion of the dislocations (present in the simulations and absent in the experiments), the matching of simulations and experiments is hampered even in the case of a dc in-plane magnetic field-driven motion. The reason is that, in the simulations, pinning centers have not been included; whereas, in the real Fe-N film, ever-present and randomly distributed structural defects act as pinning centers which affect, and even stop, the field-driven motion of magnetic edge dislocations.

### Theoretical model

#### The Thiele equation

To gain further insight into the field-induced displacement of magnetic edge dislocations, approximate analytical calculations using the Thiele equation^37–39^

1 $${{\bf{F}}}_{{\rm{tot}}}={\rm{0}}={{\bf{F}}}_{{\rm{ext}}}+{{\bf{F}}}_{{\rm{gyro}}}+{{\bf{F}}}_{{\rm{diss}}}=-\,\nabla {U}+{\bf{G}}\times {\bf{v}}+{\mathscr{D}}\times {\alpha }{\bf{v}}$$

were performed. Equation 1 is a reformulation of the LLG equations, where a steady-state motion (i.e., ${{\bf{F}}}_{{\rm{tot}}}=0$) is assumed^37,38^ for the magnetic domains of a given magnetization distribution, ${\bf{n}}={\bf{M}}/{M}_{s}$. As deformations of the magnetic texture are not allowed during the motion at constant velocity *v*, the internal forces (due to exchange coupling, magnetostatic interactions and magnetic anisotropy energy) must not^37,38^ be included explicitly in (1). In our case, **F**_*ext*_ is the external force on a magnetic edge dislocation, generated by the Zeeman potential energy (*U*) of the applied dc magnetic field; ${{\bf{F}}}_{{\rm{gyro}}}={\bf{G}}\times {\bf{v}}$ (where **G** is the gyrovector associated with **n**) is the gyrotropic force, pushing the defect perpendicular to its direction of motion; ${{\bf{F}}}_{{\rm{diss}}}={\bf{D}}\cdot \alpha {\bf{v}}$ is the dissipative force (where ${\mathscr{D}}$ is the dissipation dyadic tensor associated with **n**, and *α* > 0 is the Gilbert damping constant), hindering the motion of the defect without changing its direction.

#### Magnetization configuration

**Figure 6:**
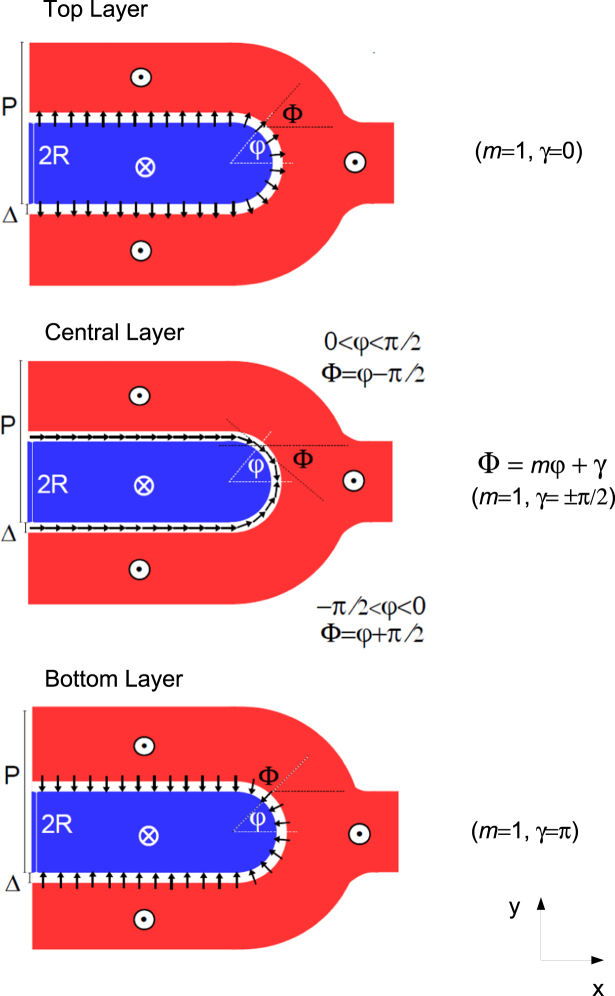
Idealized magnetization distribution of a magnetic edge dislocation across film thickness. Sketch of the idealized static magnetization distribution of a magnetic edge dislocation at ${H}\simeq 0$, for the top, central and bottom film layer, respectively, which was assumed in order to calculate analytically the forces in the Thiele Eq. (1). Red, white and blue regions have *n*_*z*_ > 0, *n*_*z*_ = 0 and *n*_*z*_ < 0, respectively. In the semicircular transition region with diameter 2*R* = *P*/2 and width ${\rm{\Delta }}\ll {R}$, the in-plane magnetization (black arrows) configuration, characterized by the angle Φ = *mφ* + *γ*, was assumed to have constant vorticity (*m* = 1), but different helicity $({\gamma }=0,\pm \frac{{\rm{\pi }}}{2},{\pi })$ depending on the film layer position along *z*.

**Figure 7:**
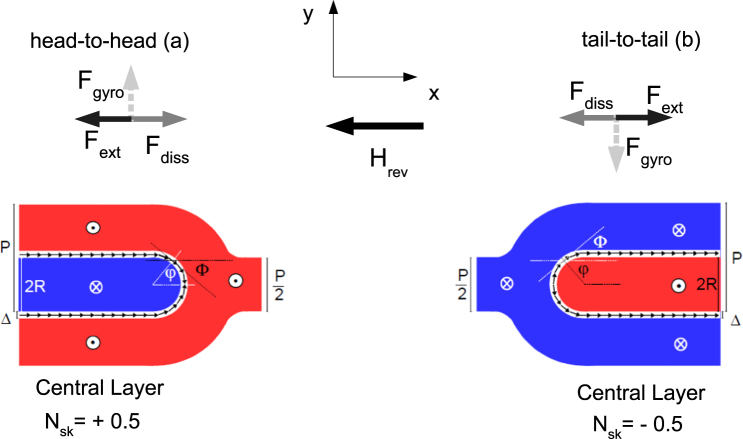
The forces acting on a pair of magnetic edge dislocations for moderate reversal field. Sketch of the external, dissipative and gyrotropic force acting on a pair of opposite magnetic edge dislocations, supposed to perform a stationary motion according to the Thiele equation (Eq. 1). A sufficiently small dc magnetic field, **H**_rev_, is applied antiparallel to the stripes axis, **e**_*x*_. The external force, **F**_ext_, is directed along the stripes axis and changes its sign depending on the (idealized) in-plane magnetization distribution in the central film layer of the dislocation, whether head-to-head (**a**) or tail-to-tail (**b**). For magnetic edge dislocations embedded in a periodic stripe domain pattern, the gyrotropic force, **F**_gyro_, vanishes (dashed arrow). Consequently, the dissipative force, **F**_diss_, is equal and opposite to **F**_ext_. All forces reverse their direction when the driving magnetic field is reversed.

For the sake of simplicity, the idealized magnetization configurations in Fig. 6 (respectively for the top, central and bottom region of the film) were assumed, in the presence of a dc magnetic field applied along the stripes axis (**e**_*x*_) and sufficiently small (*H* < *H*_*c*_). Moreover, the thicknesses of the top, bottom and central layers were approximately taken to be ${t}_{{\rm{top}}}={t}_{{\rm{bottom}}}=\delta$ and ${t}_{{\rm{central}}}=(t-2\delta )=t^{\prime}$. In Fig. 6, red and blue colours denote domains with opposite out-of-plane magnetization components, *n*_*z*_ > 0 and *n*_*z*_ < 0 respectively, while the white transition region in-between (a semicircumference with diameter 2*R* = *P*/2) has *n*_*z*_ = 0. The small black arrows denote in-plane magnetization vectors, arranged in a different configuration depending on the film layer in order to minimize the total energy. The normalized magnetization is expressed as

2 $${\bf{n}}({{n}}_{{x}},{{n}}_{{y}},{{n}}_{{z}})=[\sin \,{\rm{\Theta }}({r})\,\cos \,{\rm{\Phi }}({\varphi }),\,\sin \,{\rm{\Theta }}({r})\,\sin \,{\rm{\Phi }}({\varphi }),\,\cos \,{\rm{\Theta }}({r})],$$

where ${\bf{r}}=(r\,\cos \,\varphi ,\,r\,\sin \,\varphi )$ is the position vector of **n** in the film plane, *xy*; Θ(*r*) is the canting angle formed by **n** with the film normal, **e**_*z*_; Φ(*φ*) is the azimuthal angle formed by the in-plane magnetization, ${{\bf{n}}}_{{\rm{IP}}}=({n}_{x},{n}_{y})$, with the stripes axis, **e**_*x*_. For the two angles Θ(*r*) and Φ(*φ*), we assumed the analytical expressions

3 $${\rm{\Theta }}({r})=\frac{\pi }{2}[1+{p}\,\tanh (\frac{{r}-{R}}{{\rm{\Delta }}})],\,{\rm{\Phi }}({\varphi })=m{\varphi }+{\gamma },$$

where $p=\pm \,1$ is the *z*-polarization parameter^40^ and Δ is the width of the transition region between opposite domains; the parameter *m* = ±1 and the angle *γ* are the vorticity and the helicity of the in-plane magnetization^27,40^, respectively. The in-plane magnetization distribution in a magnetic edge dislocation was assumed to have all vectors diverging from the center of the semicircumference (i.e. *γ* = 0) in the top film region, and converging to the center (i.e., *γ* = *π*) in the bottom one. For the central film region, we assumed the in-plane magnetization, **n**_IP_, to be tangential to the semicircumference, with either a head-to-head configuration (Fig. 7a) or a tail-to-tail one (Fig. 7b). The magnetization distribution is characterized by a constant vorticity (*m* = 1), while the helicity is supposed to change from $+\frac{\pi }{2}$ (counter-clockwise) to $-\frac{\pi }{2}$ (clockwise) at the vertex of the dislocation due to the presence of a Bloch point. Now we note that, for all the three configurations, the in-plane magnetization distribution of the defect can be associated with a quantity, ${N}_{sk}=-\,\frac{1}{4\pi }\int dx\int dy\,{\bf{n}}\cdot [(\partial {\bf{n}}/\partial x)\times (\partial {\bf{n}}/\partial y)]$, named topological number^27^ (or, equivalently, topological charge^41^). In the case of constant vorticity, $m=[\frac{d{\rm{\Phi }}}{d\varphi }]$, along the semicircumference, the topological number turns out to be nonzero and half-integer.

4 $$\begin{array}{rcl}{{N}}_{{sk}} & = & \frac{1}{4{\pi }}\,{\int }_{{\rm{semicirc}}}\,d{\varphi }[\frac{{d}{\rm{\Phi }}({\varphi })}{d{\varphi }}]\,{\int }_{0}^{2{R}}\,{dr}[-\sin \,{\rm{\Theta }}({r})\frac{{d}{\rm{\Theta }}({r})}{{dr}}]\\  & = & \frac{1}{4{\pi }}({\pi }m){[\cos \,{\rm{\Theta }}({r})]}_{{r}=0}^{{r}=2{R}}=\frac{{m}}{4}(\,-\,2{p})=-\,\frac{{mp}}{2}=\pm \,\frac{1}{2}\end{array}$$

Note that the sign of *N*_*sk*_ depends on the product of the vorticity, *m*, and the *z*-polarization, *p*, while it is independent of the helicity, *γ*. For both the top and bottom region, the magnetization configuration is characterized by *m* = 1 and *p* = −1 and is therefore associated with ${{N}}_{{sk}}=+\frac{1}{2}$ (half-integer topological charge). For the central region, instead, *N*_*sk*_ depends on the configuration of the in-plane magnetization component. E.g., for the head-to-head configuration in Fig. 7a, with ${\varphi }\in [-\frac{{\rm{\pi }}}{2},+\frac{{\rm{\pi }}}{2}]$, one has *m* = 1, *p* = −1, and ${{N}}_{{sk}}=+\frac{1}{2}$; for the tail-to-tail one in Fig. 7b, with ${\varphi }\in [+\frac{1}{2}{\rm{\pi }},+\frac{3}{2}{\rm{\pi }}]$, one has *m* = 1, *p* = 1, and ${{N}}_{{sk}}=-\,\frac{1}{2}$.

Hereafter, we summarize the results obtained for the three forces in the Thiele Eq. (1) under the hypothesis of the idealized magnetization distribution in Fig. 6. Details of the analytical calculations can be found in the Supplementary Information (Sections III and IV).

#### External force

The external force, **F**_ext_, exerted on a given magnetic edge dislocation by a dc magnetic field, applied either antiparallel (**H**_rev_ = *H*_*x*_**e**_*x*_, with *H*_*x*_ < 0) or parallel (**H** = H_*x*_**e**_*x*_, with *H*_*x*_ > 0) to the stripes axis, **e**_*x*_, is given by

5 $${{\bf{F}}}_{{\rm{ext}}}=-\,{{H}}_{{x}}{{M}}_{{s}}\,{\int }_{0}^{{t}}\,{dz}\,{\int }_{{\rm{semicirc}}}\,d{\varphi }\,{\int }_{0}^{\infty }\,{rdr}\,\nabla [\sin \,{\rm{\Theta }}({r})\cos \,{\rm{\Phi }}({\varphi })]$$

where the integration range spans the whole film volume. Note that the expression (5) contains trigonometric functions of the polar angles (3). The first angle, Θ(*r*), does not depend on the film layer. The other angle, Φ(*φ*) = *mφ* + *γ*, instead, changes from layer to layer owing to the different helicity, *γ*. Therefore, the calculated **F**_ext_ (see Supplementary Information, Section V) can be separated into different contributions from different film regions (top, central, and bottom). For ${\rm{\Delta }}\ll {R}$, one has

6 $$\begin{array}{ll}{{\bf{F}}}_{{\rm{ext}},{\rm{central}}}\propto \{\begin{array}{l}+{t}^{\prime} {\rm{\Delta }}\,{{M}}_{{s}}{{H}}_{{x}}\,{{\rm{e}}}_{{x}}\,(\mathrm{head}-{\rm{to}}-\mathrm{head})\\ -{t}^{\prime} {\rm{\Delta }}\,{{M}}_{{s}}{{H}}_{{x}}\,{{\rm{e}}}_{{x}}\,(\mathrm{tail}-{\rm{to}}-\mathrm{tail})\end{array} & {{\bf{F}}}_{{\rm{ext}},{\rm{top}}}={{\bf{F}}}_{{\rm{ext}},{\rm{bottom}}}\approx {\rm{0}}\end{array}$$

Namely, only a subset of the total film (with thickness *t*′ < *t*), where approximately the same magnetization configuration as in the central film layer is realized, provides a nonzero **F**_ext_. The top and bottom film layers, where flux-closure domains are present, do not contribute to **F**_ext_. However these layers, being strongly exchange-coupled to the central region, participate into the rigid motion of the dislocation. Note that, in Eq. 6, the calculated **F**_ext_ does not explicitly depend on the z-polarization, *p*. The external force turns out to be directed along the stripes axis, **e**_*x*_, and reverses its direction according to the in-plane magnetization distribution in the central film layer. For reversal field (*H*_*x*_ < 0), the forces are schematically depicted in Fig. 7, under the assumption of a head-to-head (Fig. 7a) or a tail-to-tail (Fig. 7b) configuration. For positive field (*H*_*x*_ > 0), **F**_ext_ reverses its direction (see Supplementary Information, Section IV). A final consideration should be made about the idealized magnetization configurations (head-to-head and tail-to-tail) we assumed for the analytical calculation of **F**_ext,central_ in Eq. 6. In the Supplementary Information (Section VI), other in-plane magnetization configurations of the central film layer were considered, and detailed analytical calculations provided a zero external force, **F**_ext_ = 0, for configurations without a Bloch point at the end of the stripe. In contrast, configurations with a Bloch point at the end of the stripe were found to be driven by a dc in-plane magnetic field. Noticeably, the two idealized configurations associated with nonzero external force, **F**_ext_ ≠ 0, are characterized by different topological numbers (*N*_*sk*_) but share a common property: the product (*mγ*) of vorticity, *m*, and helicity, *γ*, changes sign at the vertex of the dislocation. It is worth noting that similar magnetization configurations, with a Bloch point at the end of the stripe, were found^18,19^ to play an important role in the magnetization reversal process of stripe domain patterns in magnetic films and multilayers. In these materials, the weak PMA determines significant variations in the magnetization configuration along the film normal^18,19^, which could be measured using x-ray magnetic microscopy. The same imaging technique, complemented by micromagnetic calculations, was exploited in the study of the in-plane magnetization inversion at the bifurcations of the stripe domain pattern in magnetic trilayers^20^. It might, thus, prove useful for the purpose of obtaining a quantitative experimental check for the hypothesized magnetization configurations of the magnetic edge dislocations even in the case of our Fe-N film.

#### Gyrotropic force

The gyrotropic force, **F**_gyro_ = **G** × **v**, acting on a magnetic edge dislocation, is expressed in terms of the gyrovector **G** (*γ*_*e*_ is the electron gyromagnetic factor)

7 $$\begin{array}{rcl}{\bf{G}} & = & -\frac{{{M}}_{{s}}}{{{\gamma }}_{{e}}}\,\int \,{dz}\,\int \,{d}{\varphi }\,\int \,{rdr}\,\sin \,{\rm{\Theta }}({r})[{\nabla }_{{\bf{r}}}{\rm{\Theta }}({r})\times {\nabla }_{{\bf{r}}}{\rm{\Phi }}({\varphi })]\\  & = & \frac{{{M}}_{{s}}}{{{\gamma }}_{{e}}}\,{\int }_{0}^{{t}}\,{dz}\,{\int }_{{\rm{semicirc}}}\,{d}{\varphi }\,[\frac{{d}{\rm{\Phi }}({\varphi })}{d{\varphi }}]\,{\int }_{0}^{\infty }\,{dr}[-\sin \,{\rm{\Theta }}({r})\,\frac{{d}{\rm{\Theta }}({r})}{{dr}}]\,{{\bf{e}}}_{{z}}\end{array}$$

where, as in Eq. 5, the integration spans the whole film volume. However, we note that an explicit calculation of **F**_gyro_ is not necessary because, even if a magnetic edge dislocation is characterized by a nonzero topological number (cfr. Eq. 4, where ${{N}}_{{sk}}=\pm \frac{1}{2}$), when the defect is embedded in a periodic stripe domain pattern, the gyrovector is found to vanish on the basis of purely topological considerations.

8 $$\begin{array}{ccc}{\bf{G}}=0, & {{\bf{F}}}_{{\rm{gyro}}}={\bf{G}}\times {\bf{v}}=0 & ({\rm{defect}}\,{\rm{embedded}}\,{\rm{in}}\,{\rm{a}}\,{\rm{stripe}}\,{\rm{pattern}})\end{array}$$

Indeed, in (7), the *r*-integration is extended from 0 to ∞. Therefore, for any film layer (i.e., for any fixed *z*) the result of the double integration (on *r* and *φ*) is proportional to the topological number of a periodic stripe domain pattern, which is *N*_*sk*_|_stripes_ = 0. It follows that **G** and **F**_gyro_ are both zero, as far as a well-defined periodic stripe domain pattern is preserved in the film (i.e., for sufficiently low values of the driving dc magnetic field).

#### Dissipative force

First, we note that the contribution to the dissipative force, **F**_diss_, is the same for each film layer because (see Supplementary Information, Section IV) the expression of the dissipation dyadic ${\mathscr{D}}$ does not involve the helicity angle, *γ*. For a translational motion of the magnetization pattern parallel to the stripes axis, with velocity **v** = *v*_*x*_**e**_*x*_, the dissipative force is found to be

9 $${{\bf{F}}}_{{\rm{diss}}}=-\,\alpha |{{\mathscr{D}}}_{{xx}}|{{v}}_{{x}}{{\bf{e}}}_{{x}}$$

where *α* > 0 is the Gilbert damping parameter. Therefore, **F**_diss_ has just the effect of hindering the translational motion of the dislocation along the stripes axis. For a defect embedded in a stripe pattern (**F**_gyro_ = 0, see Eq. 8), it turns out that the dissipative force, **F**_diss_, is equal and opposite to the external force, **F**_ext_ (cfr. Eq. 1, where the condition **F**_tot_ = 0 for steady-state motion was assumed at the outset).

Summing up, making use of the Thiele equation and assuming an approximate magnetization distribution for magnetic edge dislocations at remanence, or for very small dc magnetic field applied along the stripes axis, we have found that:

- The gyrovector **G** and the gyrotropic force **F**_gyro_ both vanish for a magnetic edge dislocation embedded in a stripe domain pattern, even if the defect carries a nonzero half-integer topological charge, ${{N}}_{{sk}}=\pm \frac{1}{2}$, in some layers across the film thickness.
- The driving esternal force, **F**_ext_, and the hindering dissipative force, **F**_diss_, both directed along the stripes axis, are equal and opposite. Moreover, the direction of the external force is only determined by the in-plane magnetization distribution of the magnetic edge dislocation in the central region of the film.
- All the forces reverse their direction when the magnetic field is reversed.

The above theoretical results appear to be consistent with the main feature observed both in MFM experiments and numerical simulations: namely, the straight displacement of a pair of magnetic edge dislocations into opposite directions along the stripes axis in the presence of a dc magnetic field, small enough to preserve the stripe domain structure.

## Discussion

In summary, we have investigated the motion of magnetic edge dislocations in an ${{\rm{N}}}_{2}^{+}$-implanted Fe film with a stripe domain pattern by combining MFM data, micromagnetic simulations and a theory based on the approximate Thiele equation. When a moderate dc magnetic field is applied in plane along the stripes axis, the displacement of the defects has been found to occur either parallel or antiparallel to it, depending on the distribution of the in-plane magnetization in the dislocations. Our theoretical analysis has shown that, even if the magnetic edge dislocation is characterized by a nonzero, half-integer topological number, the gyrovector associated with the magnetization distribution in the defect vanishes (**G** = 0) owing to the presence of the periodic stripe domain pattern, and as a consequence also the gyrotropic force on the magnetic edge dislocation vanishes (**F**_gyro_ = 0). In conclusion, our theoretical analysis suggests that the topological properties of magnetic edge dislocations embedded in a stripe domain pattern are fundamental to explain why the displacement of dislocations takes place along straight trajectories, as observed both in MFM measurements and in micromagnetic simulations, for moderate intensities of a dc magnetic field applied along the stripes axis: i.e., as far as the stripe domain pattern is well preserved. For chiral spin systems, a similar link between topology and dynamics^27^ was invoked to explain a somewhat opposite phenomenon: namely, the current-induced transverse deflection of a skyrmion (“skyrmion Hall effect”), which has recently been investigated both theoretically^27,42,43^ and experimentally^26,44,45^ in a magnetic track. Note, however, that in the case of our Fe-N film there is no physical patterning: rather, the field-induced displacement of magnetic edge dislocations along straight tracks is related to the very existence of a well-defined magnetic stripe domain pattern. To this regard, another interesting property of Fe-N films^22,46^ is worth mentioning: an almost rigid stripe pattern rotation can be achieved simply by saturating the magnetization along a given in-plane direction, and subsequently removing the field. As a consequence of this “rotatable anisotropy”^47–51^, the axis of propagation of the topological defects can be rotated in the same way. Clearly, in view of a desirable technological impact of Fe-N films with a stripe domain structure in the emerging field of spintronics, e.g. using magnetic edge dislocations as information carriers, two issues should first be investigated: namely, the possibility of controlled generation and spin-polarized current-driven motion of these defects.

## Methods

### Sample preparation

The Fe-N sample was prepared by implantation of nitrogen ions into an iron thin film protected against oxidization by a gold capping layer. Ion implantation (3 × 10^16^ ${{\rm{N}}}_{2}^{+}$ ions/cm^2^ at 26 keV) was carried out using the SCALP facility at Univ. Paris-Sud and CNRS/IN2P3, Université Paris-Saclay, France. The iron film was deposited on a ZnSe/GaAs(001) substrate and capped with gold in a molecular beam epitaxy (MBE) chamber. The iron epitaxial growth was performed with the following in-plane orientations: *α*-Fe[110]|| ZnSe[110]|| GaAs[110] and *α*-Fe(001)|| ZnSe(001)|| GaAs(001), as testified by *in situ* reflection high-energy electron diffraction (RHEED) measurements. ZnSe is used as a chemical barrier to separate the iron layer from the semiconductor substrate. The thicknesses of the iron thin film and its gold capping layer, which are 78 nm and 8 nm respectively, were determined by x-ray reflectometry (XRR). The iron thin film was implanted with ${{\rm{N}}}_{2}^{+}$ ions at an energy of 26 keV with a fluence of 3 × 10^16^ ions/cm^2^ at room temperature. The implantation was done without cooling system for the target; besides, the ion current density was kept at about 5 *μ*A/cm^2^. A normally incident ion beam was used, the gold capping layer preventing channeling effect in the iron film. Remarkably, the nitrogen concentration presents a homogeneous distribution in the thin film, as attested by Rutherford backscattering spectrometry (RBS) and Elastic Recoil Detection Analysis (ERDA) experiments using the 43 MeV Cl^7+^ ion beam facility in Helmholtz-Zentrum Dresden-Rossendorf, Germany.

### Magnetic Force Microscopy measurements

In-field MFM images were recorded by a Digital Instruments Nanoscope using the phase detection mode. The cantilever phase of oscillation has been monitored while the magnetic tip was scanning the sample surface at a distance of 35 nm (lift mode)^52,53^. Commercially available ferromagnetic CoCr-coated tips, magnetized to be at north pole, were used. In order to exclude the influence of the tip on the magnetic state of the sample, we tried different scanning directions and tip to sample distances, obtaining the same results with different operating conditions. From the fast Fourier transform of the images, the period of the stripe pattern has been found to be *P* = 101 nm. The in-plane magnetic field has been applied along the [−1, 1, 0] crystallographic direction. MFM measurements were performed without removing the sample from the magnetic field region, in order to analyze the changes in the domain structure of a specific area, caused by variations of the field intensity^54,55^. During the MFM measurements we observed negligible drift effects, carefully monitored by AFM and in case compensated by small displacements of the scanned area, in order to be sure of exploring the same region of the sample at every field.

### Micromagnetic simulations of the motion of magnetic edge dislocations

Micromagnetic simulations were carried out by the open-source, GPU-accelerated software MuMax3^36^. The total simulated area has dimensions of 2000 × 2000 × 78 nm^3^, and was discretized into cells having dimensions of 5 × 5 × 5.2 nm^3^. To allow an easier comparison with theory, in-plane open boundary conditions were used in the simulations (with an opportune implementation of the MuMax3 code^36^ to account for demagnetization effects at the film sides). The following parameters, obtained from FMR characterization, were used: saturation magnetization *M*_*s*_ = 1700 emu/cm^3^, exchange constant *A*_*ex*_ = 2.16 × 10^−6^ erg/cm, out-of-plane anisotropy constant *K*_*u*_ = 4.9 × 10^6^ erg/cm^3^. The sample was initially saturated applying a strong in-plane magnetic field, *H* = 4 kOe, along the [−1, 1, 0] crystallographic direction, then the intensity was reduced with a step of 100 Oe. We found that, if the magnetic field is applied strictly within the film plane, magnetic edge dislocations do not develop at remanence. Therefore, in order to promote the formation of edge defects in the stripes pattern, in our simulations the magnetic field has been tilted with respect to the surface plane by an angle, *β*, ranging typically from 1° to 3°. It is worth noticing that a similar result was obtained in a recent study^56^ of the magnetic phase diagram of Co and CoRu alloy thin films with perpendicular magnetic anisotropy.

## Electronic supplementary material

Supplementary Information

Supplementary Movie
