# Supplementary material for: Straight motion of half-integer topological defects in thin Fe-N magnetic films with stripe domains
Source: Sci Rep. 2018 Jun 19;8:9339. doi: 10.1038/s41598-018-27283-7 (PMC6008308; doi:10.1038/s41598-018-27283-7)
Supplement: Supplementary file 1 — Supplementary Information [file 41598_2018_27283_MOESM1_ESM.pdf]

Supplementary Information for the article:
"Straight motion of half-integer topological defects in thin Fe-N magnetic films with stripe domains"

S. Fin,[1] R. Silvani,[2] S. Tacchi,[3] M. Marangolo,[4] L. C. Garnier,[4,5] M. Eddrief,[4]
C. Hepburn,[4] F. Fortuna,[6] A. Rettori,[7] M. G. Pini,[8,*] and D. Bisero[1,9,†]

[1] *Dipartimento di Fisica e Scienze della Terra, Università degli Studi di Ferrara, Via Saragat 1, I-44122 Ferrara, Italy*
[2] *Dipartimento di Fisica e Geologia, Università di Perugia, I-06123 Perugia, Italy*
[3] *Istituto Officina dei Materiali del CNR (CNR-IOM), Unità di Perugia, c/o Dipartimento di Fisica e Geologia, Università di Perugia, I-06123 Perugia, Italy*
[4] *Sorbonne Université, CNRS, Institut des NanoSciences de Paris, UMR 7588, F-75252 Paris, France*
[5] *Université Versailles St-Quentin, LISV, Bâtiment Boucher, Pôle scientifique et technologique de Vélizy, 10-12 avenue de l'Europe, F-78140 Vélizy, France*
[6] *CSNSM, Université Paris-Sud and CNRS/IN2P3, Université Paris-Saclay, F-91405 Orsay, France*
[7] *Dipartimento di Fisica ed Astronomia, Università di Firenze, I-50019 Sesto Fiorentino (FI), Italy*
[8] *Istituto dei Sistemi Complessi del CNR (CNR-ISC), Unità di Firenze, I-50019 Sesto Fiorentino (FI), Italy*
[*] email: mariagloria.pini@isc.cnr.it
[9] *CNISM, Unità di Ferrara, I-44122 Ferrara, Italy*
[†] email: bisero@fe.infn.it

(Dated: May 22, 2018)

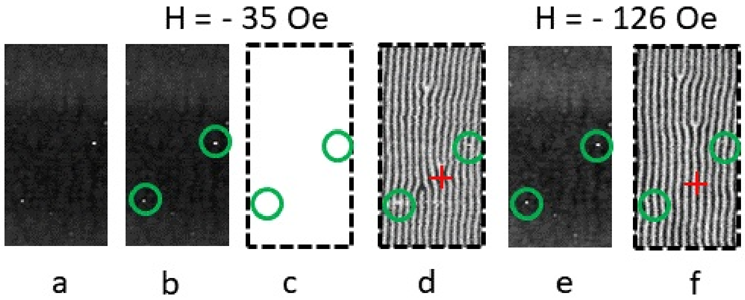

FIG. 1. **Schematic description of the method used to measure the displacement of magnetic edge dislocations.** a) AFM image at a reversal field near remanence ($H = -35$ Oe): two main particles are clearly visible; b) we mark them with green circles on the figure and note down their exact surface coordinates; c) we create an empty mask, where we report only the green circles in the positions determined in b); d) we superimpose the mask in c) on the MFM image; now we can choose a particular dislocation and mark it with a red cross; e) we apply a magnetic field $H = -126$ Oe and measure the same area, where the two particles are still present in the AFM image; we repeat the procedure described from a) to d) and obtain figure f); f) the red cross is maintained in the same position of figure d), as deduced from the coordinates of the green circles, showing that the dislocation has now moved with respect to the red cross.

I. EXPERIMENT: DESCRIPTION OF THE METHOD USED TO MEASURE THE POSITION OF MAGNETIC EDGE DISLOCATIONS

Topographic imperfections are generally considered a bother in the Magnetic Force Microscopy (MFM) technique but, in our experiment, they were a help to create a map of the analyzed surface. It is well known that the adhesion of fine particles of contaminants on the surface of a sample measured in air is unavoidable. For this reason we protected our Fe-N film with a gold capping layer. In this way, the particles (maximum size below 10 nm) can be detected during the Atomic Force Microsopy (AFM) scan, but they do not affect the MFM results, since the lift height of the magnetic analysis was well above 30 nm. In our measurements we marked the particles present on the gold surface, detected by AFM, and, superimposing the relative MFM image, we transferred the markers position on the stripes. This procedure was repeated for all MFM images taken at different fields, referring to the same archipelago of particles, that did not move from one measurement to the other. In this way, one can assure that the analyzed area is the same at every field and can deduce the precise position of the magnetic edge dislocations with respect to the topography, investigating their evolution and motion in the presence of a magnetic field. A schematic example of the technique is reported in the figure 1. This is just an example, demonstrating how with this procedure one can get exact coordinates of all the points included in the examined area, starting from the position of surface particles, and transfer the deduced map on the MFM image. This allowed us to follow the motion of the magnetic dislocations, occurring under the protecting gold layer, in the Fe-N film.

II. EXPERIMENT: EFFECT OF REVERSING AN IN-PLANE APPLIED MAGNETIC FIELD ON MFM DATA TAKEN AT REMANENCE.

Here we present some supplementary MFM data, taken on the same Fe-N sample as the one used to obtain the MFM data in Fig. 1 of the Main Article. It is important to note that all the supplementary MFM images in Fig. 2 have been taken at remanence, using a different experimental apparatus located in Paris. In the supplementary MFM images, blue rectangles and squares denote corresponding areas of the sample surface, while blue crosses mark topographical defects taken as landmarks (using the method described in the previous Section). Green and red circles denote the positions of two different types of magnetic edge dislocations, while green and red arrows denote the direction of their motion after the application of an in-plane magnetic field H_{app}. We remind that the field was removed before taking each MFM image. Note that, in Fig. 2, a positive in-plane magnetic field was applied in the North-East (NE) direction, while a negative field in the South-West (SW) direction.

The sample was first saturated in plane by applying a strong positive magnetic field (H_{app}=5000 Oe), then the

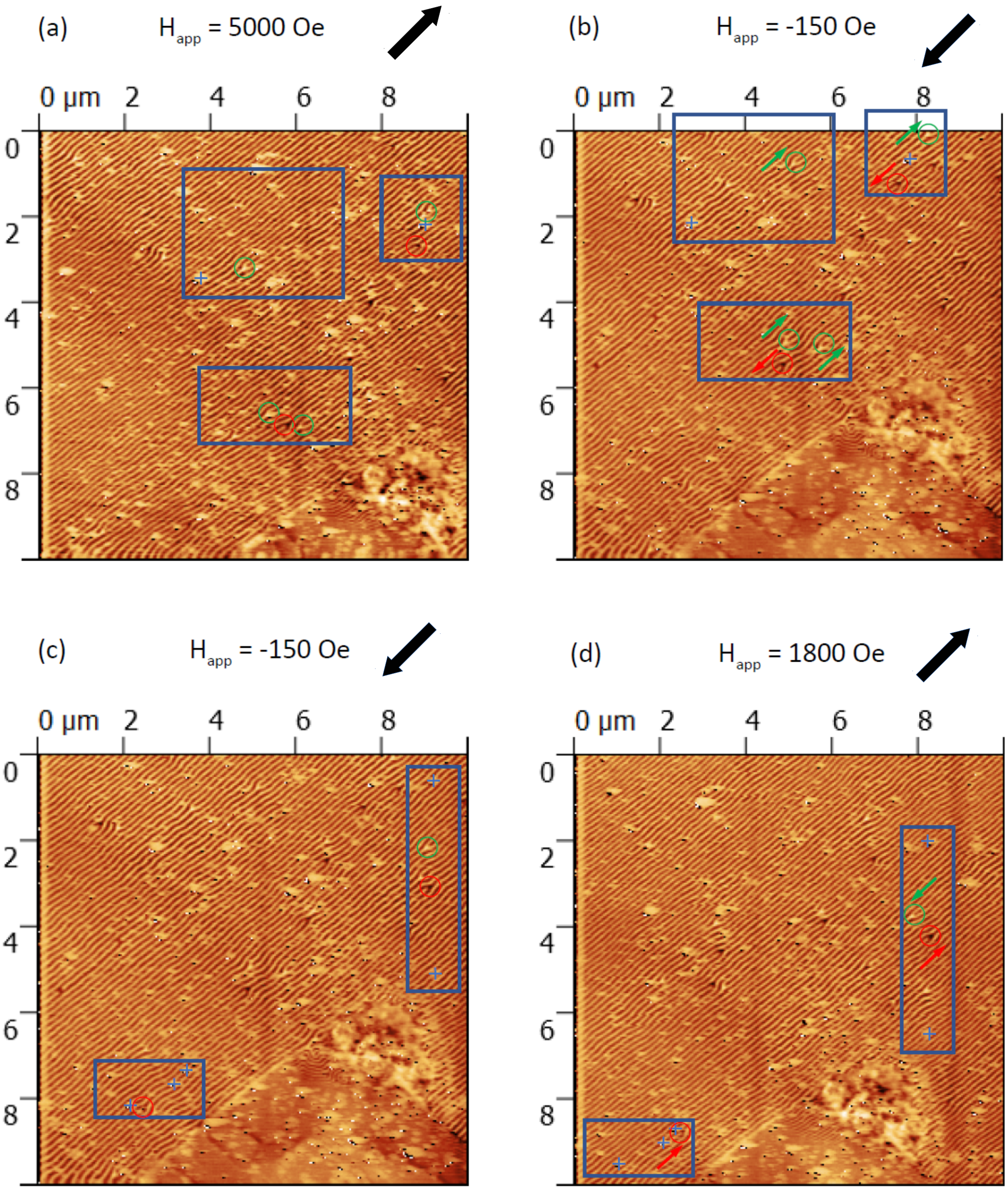

FIG. 2. **Effect of reversing an in-plane applied magnetic field on MFM data taken at remanence.** MFM images of the Fe-N film taken at remanence after a magnetic field H_{app}, of variable intensity and sign in the various panels, was applied in plane. Blue rectangles and squares denote corresponding areas of the sample surface, while blue crosses mark topographical defects taken as landmarks using the method illustrated in Fig. 1. Green and red circles denote the positions of two different types of magnetic edge dislocations, while green and red arrows denote the direction of their motion after the application of the in-plane magnetic field H_{app} and its subsequent removal. A positive field was applied in plane in the North-East (NE) direction (panels (a) and (d)), while a negative field in the South-West (SW) direction (panels (b) and (c)), as indicated by the black thick arrows. One can infer that some dislocations exist, for which the inversion of the in-plane magnetic field H_{app} induces an inversion in their displacement; moreover, opposite dislocations in a pair appear to move into opposite directions.

field was removed. A stripe pattern with two types of magnetic edge dislocations, marked by red and green crosses, developed: see Fig. 2(a). Next, a negative magnetic field (H_{app}=-150 Oe) was applied and subsequently removed: see Fig. 2(b). One can observe that the "green" dislocations moved to NE and the "red" dislocations moved to SW with respect to the blue crosses (i.e., the still landmarks).

Afterwards, different regions of the sample surface were analyzed after application, and subsequent removal, of a moderate negative field: see Fig. 2(c) (H_{app}=-150 Oe). Next, the same analysis was performed after application, and subsequent removal, of a positive field: see Fig. 2(d) (H_{app}=1800 Oe). One can observe that, in this case, the "green" dislocations moved to SW and the "red" dislocations moved to NE with respect to the blue crosses.

From these MFM data taken at remanence, one can therefore infer that some dislocations exist, for which the inversion of the in-plane applied magnetic field induces an inversion in their displacement; moreover, opposite dislocations in a pair appear to move into opposite directions.

III. THEORY: EQUATIONS OF MOTION AND TOPOLOGICAL PROPERTIES

The evolution in time of a given magnetic texture, $\mathbf{n} = \mathbf{M}/M_s$, where M_s is the saturation magnetization, is phenomenologically described by the Landau-Lifshitz-Gilbert (LLG) equations of motion[1,2], possibly modified[3,4] to include the contribution of spin transfer torques[5,6]

$$\frac{d\mathbf{n}}{dt} = -\gamma_e\ \mathbf{n}\times\mathbf{H}_{\text{eff}} + \alpha\ \mathbf{n}\times\frac{d\mathbf{n}}{dt} - (\mathbf{u}\cdot\nabla)\mathbf{n} + \beta\mathbf{n}\times[(\mathbf{u}\cdot\nabla)\mathbf{n}] \tag{1}$$

where $\gamma_e = \frac{|e|}{2m_e}g_e = \frac{\mu_B}{\hbar}g_e$ is the electron gyromagnetic ratio; e, m_e and g_e are the electron charge, mass and g-factor, respectively; μ_B the Bohr magneton and $\hbar$ the Planck constant; $\mathbf{H}_{\text{eff}}$ the local effective field; $\alpha > 0$ the Gilbert damping and β the non-adiabaticity parameter[7]. The last two terms in (1) respectively represent the adiabatic[4] and non-adiabatic[5,6] contribution to the torque, acting on $\mathbf{n}$ due to an electrical current, with density $\mathbf{j}$ and spin polarization P_s; $\mathbf{u} = \frac{g_e\mu_B}{2|e|M_s}P_s\mathbf{j}$ is the velocity associated with the spin-polarized electrical current.

Starting with the LLG equations of motion (1), Thiele[8,9] derived an approximate equation[10,11] in order to describe the steady-state dynamics of a domain wall. In the presence of both an external magnetic field and a spin-polarized electrical current, the Thiele equation reads[12]

$$0 = \mathbf{F}_{\text{tot}} = \mathbf{F}_{\text{ext}} + \mathbf{F}_{\text{gyro}} + \mathbf{F}_{\text{diss}} = -\nabla U + \mathbf{G}\times(\mathbf{v}-\mathbf{u}) + \mathcal{D}\cdot(\alpha\mathbf{v} - \beta\mathbf{u}) \tag{2}$$

where $\mathbf{v}$ is the drift velocity induced by an external magnetic field, and $\mathbf{u}$ is the velocity associated with a spin-polarized electrical current. According to the Thiele equation (2), the total force, $\mathbf{F}_{\text{tot}}$, acting on the domain wall in the magnetic texture, is the sum of three terms: (i) an external force, $\mathbf{F}_{\text{ext}} = -\nabla U$; (ii) a gyrotropic force, $\mathbf{F}_{\text{gyro}} = \mathbf{G}\times(\mathbf{v}-\mathbf{u})$; (iii) a dissipative force, $\mathbf{F}_{\text{diss}} = \mathcal{D}\cdot(\alpha\mathbf{v}-\beta\mathbf{u})$. Note that, in this work, we set $\mathbf{u} = 0$ in Eq. 2 because no spin-polarized electrical current is applied to the system.

It is important to note a fundamental difference between the micromagnetic simulations and the approximate Thiele dynamics of the dislocations. The micromagnetic simulations are performed by numerically solving the LLG equations of motion for an inhomogeneous magnetization distribution (in our case, a magnetic stripe pattern with edge dislocations, generated by the procedure described in Methods: i.e., tilting the magnetic field with respect to the film surface by an angle typically ranging from 1 to 3 degrees). In Eq. 1, all the forces due to reversible effects are included in the effective field H_{eff}: both the internal forces (due to exchange energy, demagnetizating energy, and anisotropy energy) and the external forces (due to Zeeman energy, in our case). Dissipative effects are also taken into account through the Gilbert damping α. The Thiele equation of motion (2) describes, instead, the steady-state motion of an idealized inhomogeneous distribution of the magnetization, under the effect of an external force, $\mathbf{F}_{\text{ext}}$ (in our case, the external force is due to the applied magnetic field). The gyrotropic force acting on the moving magnetization distribution accounts for its topological properties. Also in the Thiele equation dissipative effects are taken into account through the Gilbert damping, but the magnetization distribution is assumed to perform a displacement, at constant velocity $\mathbf{v}$, without any dynamical deformation. Therefore, internal forces must not be included explicitly in Eq. 2.

Let us consider the driving effect of a dc external field on the magnetic edge dislocations of a regular stripe domain pattern with period P. To fix ideas, let us assume that a strong magnetic field is first applied in plane along the versor of the stripes axis, $\mathbf{e}_x$, in order to saturate the sample. Subsequently the field is decreased towards remanence, where the stripe domain pattern develops, with $\mathbf{e}_x$ as the stripes axis. Next, the field is reversed: i.e., a dc magnetic field, $\mathbf{H}_{\text{rev}}$, is applied antiparallel to $\mathbf{e}_x$. In the following, we will also consider the case of a dc magnetic field, $\mathbf{H}$, parallel to $\mathbf{e}_x$. In any case, the intensity of the driving magnetic field, externally applied to the stripe domain pattern, is supposed to be moderate: i.e., smaller than H_c, the coercive field.

The unit magnetization vector is expressed in polar coordinates as

$$\mathbf{n}(\mathbf{r}) = \big[\sin\Theta(r)\cos\Phi(\varphi), \sin\Theta(r)\sin\Phi(\varphi), \cos\Theta(r)\big] \tag{3}$$

where $\mathbf{r} = (r\cos\varphi, r\sin\varphi)$ is the position vector of $\mathbf{n}$ in the film plane, xy; $\Theta(r)$ is the canting angle formed by $\mathbf{n}$ with the normal to the film plane, $\mathbf{e}_z$; and $\Phi(\varphi)$ is the azimuthal angle, formed by the in-plane magnetization, $\mathbf{n}_{\rm IP} = (m_x, m_y)$, with the stripes axis, $\mathbf{e}_x$.

The Zeeman potential energy, U, the gyrovector, $\mathbf{G}$, and the dissipation dyadic, $\mathcal{D}$, are respectively given by[8,9,11]

$$U = -H_x M_s \int dz \int r dr \int d\varphi \sin\Theta(r)\cos\Phi(\varphi) \tag{4}$$

$$\mathbf{G} = -\frac{M_s}{\gamma_e}\int dz \int r dr \int d\varphi \sin\Theta(r)\big[\nabla\Theta(r)\times\nabla\Phi(\varphi)\big] \tag{5}$$

$$\mathcal{D} = -\frac{M_s}{\gamma_e}\int dz \int r dr \int d\varphi\big[\nabla\Theta(r)\otimes\nabla\Theta(r) + \sin^2\Theta(r)\ \nabla\Phi(\varphi)\otimes\nabla\Phi(\varphi)\big] \tag{6}$$

where the integrals are extended to the film volume.

For sufficiently small intensity of a dc magnetic field applied along the stripes axis, x, either parallel ($H_x > 0$) or antiparallel ($H_x < 0$) to the unit vector, $\mathbf{e}_x$, we put forward the hypothesis that the complex magnetic structure of a given magnetic edge dislocation can be approximated by the idealized spin configuration shown in Fig. 3 for the top, central and bottom film layers, respectively.

For the sake of simplicity, we assume the topological defect to have a semicircular shape with radius $R = P/4$. Note that, owing to presence of closure "cap" domains[13–16] near the top and bottom surfaces of the film, the magnetic configuration in the semicircular region of the dislocation varies along the film thickness.

For the canting angle, $\Theta(r)$, we assumed the following dependence

$$\Theta(r) = \frac{\pi}{2}\Big[1 + p\tanh\Big(\frac{r-R}{\Delta}\Big)\Big] \tag{7}$$

where $\Theta(r)$ is the angle formed by $\mathbf{n}$ with the normal to the film plane, $\mathbf{e}_z$. Note that, in our idealized spin configuration (Fig. 3), the angle $\Theta(r)$ was supposed to be independent of the layer. The parameter p is the z-polarization. For $p = +1$, as r increases from 0 to ∞, the angle $\Theta(r)$ increases monotonically from $\Theta = 0$ ("up" magnetization) to $\Theta = \pi$ ("down" magnetization). For $p = -1$, as r increases from 0 to ∞, the angle $\Theta(r)$ decreases monotonically from $\Theta = \pi$ ("down" magnetization) to $\Theta = 0$ ("up" magnetization). We suppose that $\Delta \ll R$ is the width of the transition region between two domains with opposite z-component of the magnetization.

For the azimuthal angle, $\Phi(\varphi)$, we assumed the following dependence

$$\Phi(\varphi) = m\ \varphi + \gamma \tag{8}$$

where $\Phi(\varphi)$ is the angle formed by the in-plane magnetization, $\mathbf{n}_{\rm IP}$, with the stripes axis, $\mathbf{e}_x$. The parameters m and γ are, respectively, the vorticity and helicity[17] of the in-plane magnetization, $\mathbf{n}_{\rm IP}$, in the transition region between domains with opposite values of m_z. One has $m = +1$ for a vortex arrangement of the in-plane magnetization, and $m = -1$ for an antivortex arrangement; the helicity angle, γ, in principle can take any value. Note that in our idealized spin configuration (Fig. 3) the same vorticity $m = +1$ was taken for all layers, while the helicity angle γ was supposed to depend on the layer.

At the top film surface, the presence of magnetic-flux-closure "cap" domains makes the in-plane magnetization vector, $\mathbf{n}_{\rm IP}$, point outward along the radius of the semicircle, namely the helicity angle is $\gamma = 0$.

At the bottom film surface, the magnetization in the magnetic-flux-closure "cap" domains is oppositely directed with respect to the top surface. Then, the in-plane magnetization vector, $\mathbf{n}_{\rm IP}$, points inward along the radius of the semicircle, namely the helicity angle is $\gamma = \pi$.

In the central film layer, closure domains are absent. Therefore, the in-plane magnetization, $\mathbf{n}_{\rm IP}$, is tangentially disposed with respect to the semicircumference: i.e., the helicity angle is $|\gamma| = \frac{\pi}{2}$. Moreover, at remanence (or for sufficiently small intensity of the dc driving magnetic field), we heuristically assume that a symmetric configuration is realized, with a constant vorticity ($m = +1$) and with a change in the sign of the helicity angle occurring just at the endpoint of the stripe domain (i.e. for $\varphi = 0$). Namely, in the central film layer, at remanence (or for sufficiently

small intensity of the dc driving magnetic field), we suppose either a head-to-head configuration or a tail-to-tail one to be realized

$$\begin{aligned} \gamma = +\frac{\pi}{2} \quad \text{for} \quad -\frac{\pi}{2} < \varphi < 0 \quad \text{and} \quad \gamma = -\frac{\pi}{2} \quad \text{for} \;\; 0 < \varphi < \frac{\pi}{2}, \quad \text{head}-\text{to}-\text{head (a)} \\ \gamma = +\frac{\pi}{2} \quad \text{for} \quad +\frac{\pi}{2} < \varphi < \pi \quad \text{and} \quad \gamma = -\frac{\pi}{2} \quad \text{for} \;\; \pi < \varphi < \frac{3}{2}\pi, \quad \text{tail}-\text{to}-\text{tail (b)} \end{aligned} \tag{9}$$

We remind that $\gamma = +\frac{\pi}{2}$ is associated with a counter-clockwise circulation of the in-plane magnetization, while $\gamma = -\frac{\pi}{2}$ is associated with a clockwise circulation. Clearly, it depends on the history of the sample whether a head-to head configuration or a tail-to-tail one is realized, for a given topology of the defect. E.g. the head-to-head configuration shown in the central panel of Fig. 3 is expected to be realized, at remanence (or for sufficiently small intensity of the driving dc magnetic field), in a film which was previously saturated in plane along $\mathbf{e}_x$.

In the following, we are going to establish a link between the dynamical behavior and the topological properties of the in-plane magnetization distribution of the defect. Namely, within a fixed film layer, the in-plane magnetization distribution in the magnetic edge dislocation can be associated with a topological number[17] (or, equivalently, a topological charge[18])

$$N_{sk} = -\frac{1}{4\pi} \int \left[\mathbf{n} \cdot \left(\frac{\partial \mathbf{n}}{\partial x} \times \frac{\partial \mathbf{n}}{\partial y} \right) \right] dxdy = \frac{1}{4\pi} \int_{\text{semicirc}} d\varphi \left[\frac{d\Phi(\varphi)}{d\varphi} \right] \int_0^{2R} dr \left[-\sin\Theta(r) \, \frac{d\Theta(r)}{dr} \right] \tag{10}$$

The φ-integration simply provides πm. Note that this result holds not only for the central film layer and for the head-to-head configuration explicitly considered on the r.h.s. of Eq. 10. Rather, it holds for any configuration of a given film layer, provided only that a constant vorticity ($m = \frac{d\Phi}{d\varphi}$) is assumed for the in-plane magnetization configuration along the semicircumference. As regards the r-integration, one has to consider that the defect is associated with a localized magnetization texture with a finite radius, R. Therefore, the integration range can be limited between 0 and $2R$. Depending on the z-polarization parameter ($p = \pm 1$), one therefore obtains that the topological number, N_{sk}, associated with the in-plane magnetization distribution of the defect in each film layer is a half integer

$$N_{sk} = \frac{1}{4\pi} (\pi m) \Big[\cos\Theta(r) \Big]_{r=0}^{r=2R} = \frac{1}{4\pi} (\pi m)(-2p) = -\frac{1}{2} m p = \pm \frac{1}{2} \tag{11}$$

The sign of N_{sk} is solely determined by the vorticity (m) and the z-polarization ($p = \pm 1$), but not by the helicity (γ) of the in-plane magnetization configuration. E.g., for the idealized in-plane magnetization distribution of Fig. 3, one has, in each film layer, $m = 1$ and $p = -1$, so that $N_{sk} = +\frac{1}{2}$ does not change from layer to layer: i.e., N_{sk} is independent of the helicity angle, γ.

IV. THEORY: CALCULATION OF THE EXTERNAL, GYROTROPIC, AND DISSIPATIVE FORCE

In this Section, we perform an explicit calculation of the external force, $\mathbf{F}_{\text{ext}}$, the gyrovector, $\mathbf{G}$ and the dissipation dyadic, $\mathcal{D}$, for a magnetic edge dislocation embedded in a stripe domain structure. The magnetization configuration in the central film plane, and the forces resulting from such a calculation, are schematically depicted in Fig. 4 in the case of a reversal magnetic field applied in the film plane along the stripes axis, and in Fig. 5 in the case of a positive magnetic field. The dc magnetic field is supposed to have such a low intensity that, in the framework of the idealized model presented in the previous section, either a head-to-head (a) or a tail-to-tail (b) magnetization configuration is assumed to be realized in the central film plane, depending on the topology of the dislocation and on the history of the film.

1. External force

Malozemoff and Slonczewski[11] provided an argument to calculate the static force, $\mathbf{F}_{\text{ext}}$, tending to displace a non-equilibrium distribution of magnetization, $\mathbf{M}(\mathbf{x} - \mathbf{X})$, in the presence of some externally applied field distribution, $\mathbf{H}_{\text{ext}}(\mathbf{x})$. $\mathbf{X}$ is a vector representing the position of the distribution (in our case, of the center of the semicircular region) and $\mathbf{x}$ is the position vector of the magnetization in the local frame of reference. A distortion will be described by a change in the functional form $\mathbf{M}(\mathbf{x})$; a displacement only by a change in $\mathbf{X}$.

The external force $\mathbf{F}_{\text{ext}} = -\partial U / \partial \mathbf{X}$ is obtained differentiating the potential energy, U, of the magnetization distribution with respect to its position, $\mathbf{X}$. Any variation δU can be expressed as a volume integral of local density

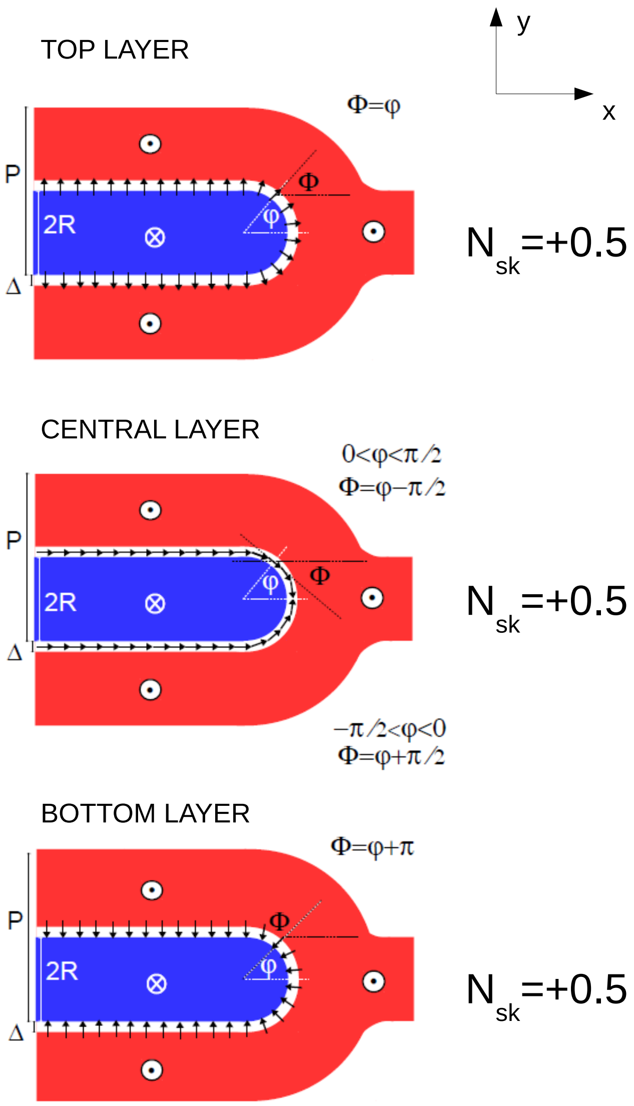

FIG. 3. **Schematic magnetization distribution of a magnetic edge dislocation along film thickness.** Sketch of the idealized magnetization distribution in a magnetic edge dislocation at remanence (or for sufficiently small intensity of the magnetic field) in the top, central and bottom film layer, respectively. Red, white and blue regions have $n_z > 0$, $n_z = 0$ and $n_z < 0$, respectively. In the semicircular transition region with diameter $2R = P/2$ and width $\Delta \ll R$, the in-plane magnetization (black arrows) is assumed to have constant vorticity ($m = 1$), while the helicity (γ) takes different values depending on the film layer. ($\gamma = 0$ in the top layer; $|\gamma| = \frac{\pi}{2}$ in the central layer; $\gamma = \pi$ in the bottom layer).

variations $\delta u(\mathbf{x} - \mathbf{X})$; therefore the external force can be written in two alternative forms[11]

$$\mathbf{F}_{\rm ext} = \int \big(-\delta u/\delta \mathbf{X}\big) dV, \qquad \mathbf{F}_{\rm ext} = \int \big(\delta u/\delta \mathbf{x}\big) dV \tag{12}$$

In the following, we use the second expression (on the right of Eq. 12). Moreover, we take into account that

$$\nabla = \frac{\partial}{\partial r}\mathbf{e}_r + \frac{1}{r}\frac{\partial}{\partial \varphi}\mathbf{e}_\varphi = \left[\cos\varphi\frac{\partial}{\partial r} - \frac{1}{r}\sin\varphi\frac{\partial}{\partial\varphi}\right]\mathbf{e}_x + \left[\sin\varphi\frac{\partial}{\partial r} + \frac{1}{r}\cos\varphi\frac{\partial}{\partial\varphi}\right]\mathbf{e}_y \tag{13}$$

where $\mathbf{e}_r = \cos\varphi\mathbf{e}_x + \sin\varphi\mathbf{e}_y$ and $\mathbf{e}_\varphi = -\sin\varphi\mathbf{e}_x + \cos\varphi\mathbf{e}_y$ respectively denote the radial and tangential unit vector.

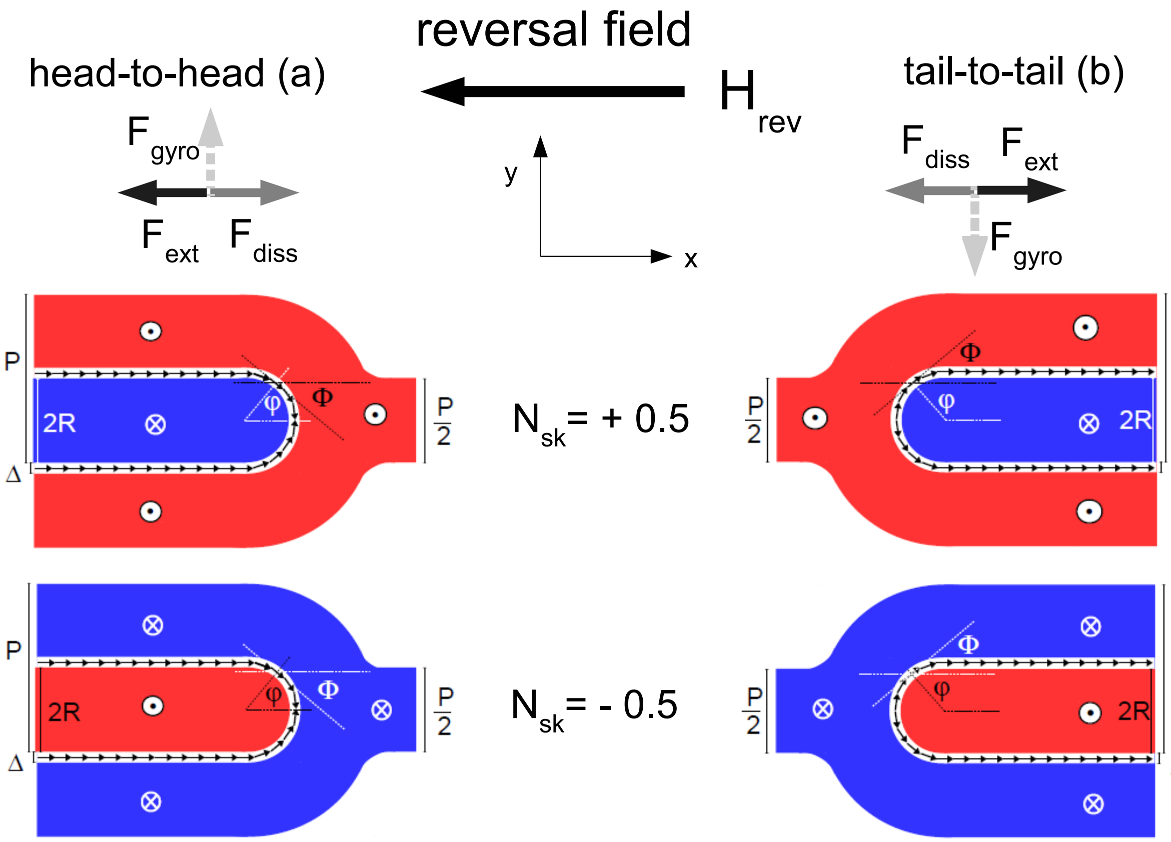

FIG. 4. **The forces acting on a magnetic edge dislocation for moderate reversal field.** Sketch of the four possible types of magnetic edge dislocation, in a stripe domain pattern with period P, when a moderate dc magnetic field, $\mathbf{H}_{\rm rev}$, is applied antiparallel to the stripes axis, $\mathbf{e}_x$. Red, white and blue regions have $n_z > 0$, $n_z = 0$ and $n_z < 0$, respectively. The small black arrows in the semicircular transition region with radius $R = P/4$ and width $\Delta \ll R$ are arranged in-plane either head-to-head (a) or tail-to-tail (b). $\mathbf{F}_{\rm ext}$, $\mathbf{F}_{\rm diss}$ and $\mathbf{F}_{\rm gyro}$ denote respectively the external, dissipative and gyrotropic force in the Thiele equation (2). $N_{sk} = \pm\frac{1}{2}$ is the topological number associated with an isolated defect.

The external force, $\mathbf{F}_{\rm ext}$, associated with a magnetic field applied along the stripes axis, $\mathbf{H} = H_x \mathbf{e}_x$, can be expressed in Cartesian coordinates as

$$\begin{aligned}\mathbf{F}_{\rm ext} =& - H_x M_s \int dz \int r dr \int d\varphi \, \nabla \Big[\sin\Theta(r) \cos\Phi(\varphi) \Big] = -H_x M_s \int_0^t dz \int_0^\infty dr \int_{\rm semicirc} d\varphi \\ &\times \Bigg\{ \left[r \cos\Theta(r) \frac{d\Theta(r)}{dr} \; \cos\Phi(\varphi) \cos\varphi + \sin\Theta(r) \; \sin\Phi(\varphi) \sin\varphi \frac{d\Phi(\varphi)}{d\varphi} \right] \mathbf{e}_x \\ &+ \left[r \cos\Theta(r) \frac{d\Theta(r)}{dr} \; \cos\Phi(\varphi) \sin\varphi - \sin\Theta(r) \; \sin\Phi(\varphi) \cos\varphi \frac{d\Phi(\varphi)}{d\varphi} \right] \mathbf{e}_y \Bigg\} \end{aligned} \tag{14}$$

where the φ-integration is extended to the semicircumference.

We now take into account, in an approximate way, the layer dependence of the azimuthal angle, $\Phi(\varphi) = m\varphi + \gamma$, by assuming the following z-dependence for the helicity, γ:

$$\gamma = \begin{cases} 0, & 0 < z < \delta \\ \pm\dfrac{\pi}{2}, & \delta < z < (t - \delta) \\ \pi, & (t-\delta) < z < t \end{cases} \tag{15}$$

In the Appendix A, we explicitated the calculation of $\mathbf{F}_{\rm ext}$, by separating the contributions from the top, bottom

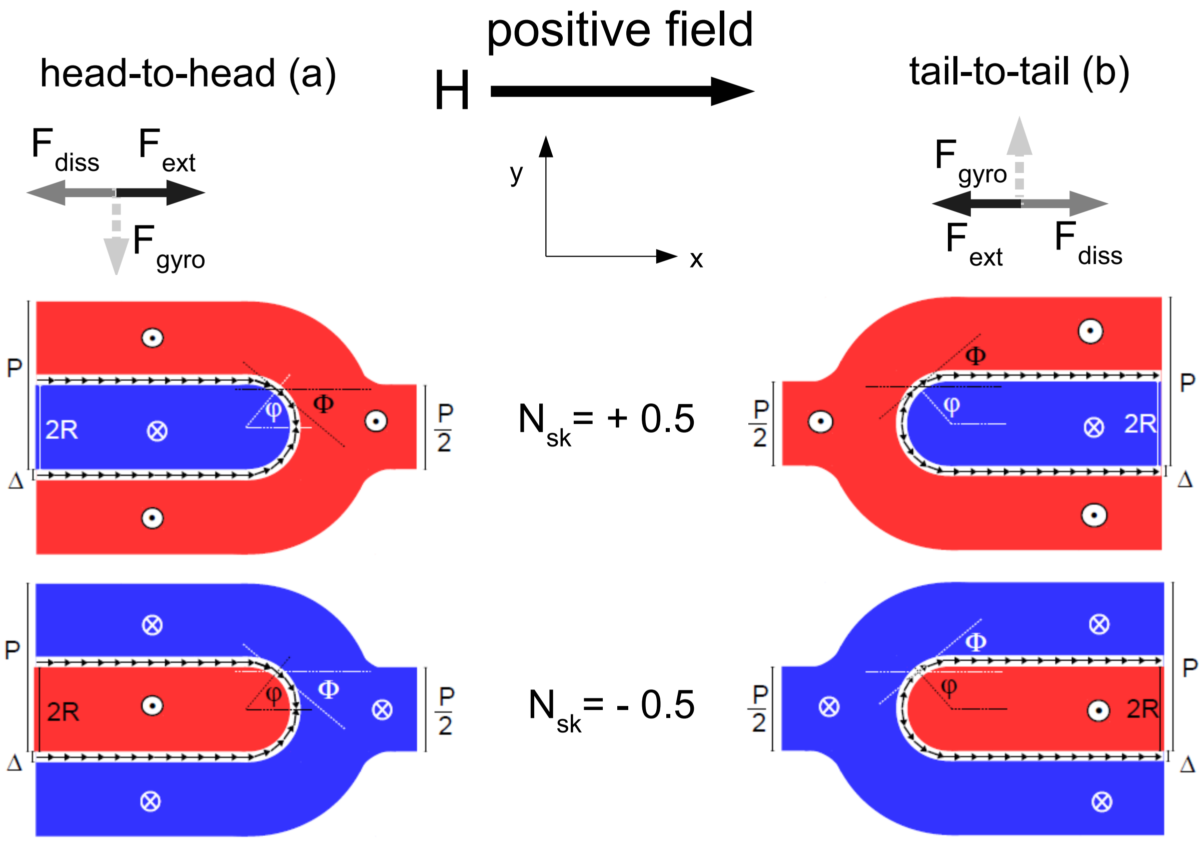

FIG. 5. **The forces acting on a magnetic edge dislocation for moderate positive field.** The same as in Fig. 4, but for a moderate field, **H**, applied parallel to the stripes axis, $\mathbf{e}_x$. Note that all the forces have reversed their direction with respect to the previous figure.

and central film layers. We found that the surface layers do not contribute to the external force

$$\mathbf{F}_{\text{ext}}\Big|_{\text{top}} = \mathbf{F}_{\text{ext}}\Big|_{\text{bottom}} = 0 \tag{16}$$

Whereas, the contribution to the external force from the central film layers in the dislocation is always parallel to the applied magnetic field for a head-to-head configuration, and antiparallel to the field for a tail-to-tail configuration

$$\mathbf{F}_{\text{ext}}\Big|_{\text{central}} = \begin{cases} +(t-2\delta)\ (2\times 1.852\ \Delta)\ M_s H_x \mathbf{e}_x & (\text{head}-\text{to}-\text{head}) \\ -(t-2\delta)\ (2\times 1.852\ \Delta)\ M_s H_x \mathbf{e}_x & (\text{tail}-\text{to}-\text{tail}) \end{cases} \tag{17}$$

where δ denotes the average thickness of the surface film layers where closure "cap" domains are present, and we have made the approximation of a domain wall width much smaller than the stripe width, $\Delta \ll 2R$.

2. *Gyrotropic force*

The gyrovector **G** is defined in Eq. 5. In the case of a magnetic edge dislocation with a semicircular shape as in Fig. 3, **G** takes the form

$$\begin{aligned} \mathbf{G} &= -\frac{M_s}{\gamma_e}\int dz \int d\varphi\ \frac{d\Phi(\varphi)}{d\varphi}\int r dr\ \sin\Theta(r)\Big[\Big(\frac{d\Theta(r)}{dr}\mathbf{e}_r\Big)\times\Big(\frac{1}{r}\mathbf{e}_\varphi\Big)\Big] \\ &= \frac{M_s}{\gamma_e}\int dz\int d\varphi\Big[\frac{d\Phi(\varphi)}{d\varphi}\Big]\int dr\Big[-\sin\Theta(r)\frac{d\Theta(r)}{dr}\Big](\cos\varphi\mathbf{e}_x + \sin\varphi\mathbf{e}_y)\times(-\sin\varphi\mathbf{e}_x + \cos\varphi\mathbf{e}_y) \end{aligned}$$

$$= \frac{M_s}{\gamma_e} \int_0^t dz \int_{\text{semicirc}} d\varphi \Big[\frac{d\Phi(\varphi)}{d\varphi}\Big] \int_0^\infty dr \Big[-\sin\Theta(r) \frac{d\Theta(r)}{dr} \Big] \mathbf{e}_z \tag{18}$$

Note that, in the third line of Eq. 18, the integrand takes the same form as in Eq. 10. However, there is a fundamental difference: now, the integration range spans the whole film volume. Therefore, in the expression (18) for $\mathbf{G}$, the r-integration is extended to ∞, and there is a z-integration over the film thickness, t.

The other fundamental feature, to be taken into account for the calculation of $\mathbf{G}$, is that the defect is embedded in a periodic stripe domain pattern. Consequently, when z and φ (i.e., the layer index and the in-plane direction) are fixed, the integrand, $\mathcal{F}(r) = [-\sin\Theta(r)\frac{d\Theta(r)}{dr}]$, is a periodic function of r oscillating between equal and opposite values. Therefore, albeit the period of the integrand is not constant, if the defect is embedded in a stripe domain pattern, the r-integration yields a vanishing gyrovector and a vanishing gyrotropic force

$$\mathbf{G} = 0, \qquad \mathbf{F}_{\text{gyro}} = \mathbf{G} \times \mathbf{v} = 0 \qquad\qquad \text{(defect embedded in a stripe pattern)} \tag{19}$$

As mentioned above, the period of $\mathcal{F}(r)$ is not constant. Rather, when $|\varphi|$ decreases from $\frac{\pi}{2}$ to 0 (i.e., when the in-plane direction changes from $\mathbf{e}_y$ to $\mathbf{e}_x$), the period monotonically increases from $4R$ (i.e., the same period as the stripe domain pattern) to ∞.

Finally, it is worth observing that the result in Eq. 19, namely the vanishing of the gyrotropic force for a magnetic edge dislocation embedded in a stripe domain pattern, is quite general: i.e., it holds irrespective of the value, m, for the vorticity and γ, for the helicity of the film layer configuration. In fact, for fixed φ and fixed z, the result (19) follows just as a consequence of the vanishing of the r-integration in Eq. 18, provided that the upper limit is properly extended to ∞.

3. Dissipative force

The dissipation dyadic tensor, $\mathcal{D}$, defined in Eq. 6, is diagonal in the polar coordinates representation of $\mathbf{n}(\mathbf{r})$

$$\mathcal{D} = \begin{bmatrix} \mathcal{D}_{rr} & 0 \\ 0 & \mathcal{D}_{\varphi\varphi} \end{bmatrix} \tag{20}$$

where, taking into account that $(\frac{d\Phi}{d\varphi})^2 = m^2 = 1$, the nonzero tensor elements take the form

$$\mathcal{D}_{rr} = -\frac{M_s}{\gamma_e} \int_0^t dz \int d\varphi \int_0^\infty dr\ r\ \Big[\frac{d\Theta(r)}{dr}\Big]^2, \quad \mathcal{D}_{\varphi\varphi} = -\frac{M_s}{\gamma_e} \int_0^t dz \int d\varphi \int_0^\infty dr\ r\ \Big[\frac{\sin\Theta(r)}{r}\Big]^2 \tag{21}$$

The dissipative force $\mathbf{F}_{\text{diss}} = \mathcal{D} \cdot \alpha\mathbf{v}$ will now be calculated in Cartesian coordinates. In the general case, $\mathbf{v} = v_x\mathbf{e}_x + v_y\mathbf{e}_y$, one has

$$\mathbf{F}_{\text{diss}} = F^x_{\text{diss}}\mathbf{e}_x + F^y_{\text{diss}}\mathbf{e}_y = \alpha(\mathcal{D}_{xx}v_x + \mathcal{D}_{xy}v_y)\mathbf{e}_x + \alpha(\mathcal{D}_{yx}v_x + \mathcal{D}_{yy}v_y)\mathbf{e}_y \tag{22}$$

where the tensor elements are given by

$$\mathcal{D}_{xx} = -\frac{M_s}{\gamma_e} \int_0^t dz \int_{\text{semicirc}} d\varphi \int_0^{2R} dr\ r \Big\{ \cos^2\varphi \Big[\frac{d\Theta(r)}{dr}\Big]^2 + \sin^2\varphi \Big[\frac{\sin\Theta(r)}{r}\Big]^2 \Big\} \tag{23}$$

$$\mathcal{D}_{yy} = -\frac{M_s}{\gamma_e} \int_0^t dz \int_{\text{semicirc}} d\varphi \int_0^{2R} dr\ r\ \Big\{ \sin^2\varphi \Big[\frac{d\Theta(r)}{dr}\Big]^2 + \cos^2\varphi \Big[\frac{\sin\Theta(r)}{r}\Big]^2 \Big\} \tag{24}$$

$$\mathcal{D}_{xy} = \mathcal{D}_{yx} = -\frac{M_s}{\gamma_e} \int_0^t dz \int_{\text{semicirc}} d\varphi \int_0^{2R} dr\ r\ \sin\varphi\cos\varphi \Big\{ \Big[\frac{d\Theta(r)}{dr}\Big]^2 - \Big[\frac{\sin\Theta(r)}{r}\Big]^2 \Big\} \tag{25}$$

Now, we observe that the limits in the φ-integration are either $-\frac{\pi}{2} < \varphi < +\frac{\pi}{2}$ or $+\frac{\pi}{2} < \varphi < \frac{3}{2}\pi$. Since the primitives are $\int d\varphi \sin^2\varphi = \frac{\varphi}{2} - \frac{1}{4}\sin(2\varphi)$, $\int d\varphi \cos^2\varphi = \frac{\varphi}{2} + \frac{1}{4}\sin(2\varphi)$, $\int d\varphi \sin\varphi\cos\varphi = \frac{1}{2}\sin^2\varphi$, by parity considerations it follows immediately that, whatever the in-plane magnetization configuration, one has $\mathcal{D}_{xx} < 0$, $\mathcal{D}_{yy} < 0$ and $\mathcal{D}_{xy} = \mathcal{D}_{yx} = 0$. Moreover, we observe that the r-integration can be limited between 0 and $2R$, because for $\Delta \ll 2R$

the integrand takes the form of a narrow peak centered at $r = R$, and the results 21 are independent of the z-polarization parameter ($p = \pm 1$). Note that, in deriving $\mathcal{D}_{\varphi\varphi}$, it was taken into account that $\frac{d\Phi}{d\varphi} = m$ and $m^2 = 1$. Therefore the results for the tensor elements are also independent of the vorticity, m, and the helicity, γ.

In the particular case of a translational motion of the magnetic edge dislocation, with velocity $\mathbf{v} = v_x \mathbf{e}_x$ parallel to the stripes axis, the dissipative force takes the form

$$\mathbf{F}_{\rm diss} = -\alpha |\mathcal{D}_{xx}| v_x \mathbf{e}_x \tag{26}$$

Since $\alpha > 0$, it results that the dissipative force $\mathbf{F}_{\rm diss}$ has always the effect of hindering the motion of a dislocation.

V. APPENDIX A: EXPLICIT CALCULATION OF THE EXTERNAL FORCE FOR THE IDEALIZED MAGNETIC CONFIGURATION IN FIG. 3

Let us explicitate the calculation of the external force $\mathbf{F}_{\rm ext}$, Eq. 14, in a few special cases, $\gamma = 0, |\frac{\pi}{2}|, \pi,$, which respectively correspond to the contributions from the top, central, and bottom layers of the film as represented in Fig. 3. We always consider the case of a configuration with vorticity $m = +1$, so that one has $\frac{d\Phi(\varphi)}{d\varphi} = 1$ in Eq. 14.

- *Top layers:* $\gamma = 0$.

This is the simplest case, where $\Phi = \phi$. The external force takes the form

$$\mathbf{F}_{\rm ext}\Big|_{\rm top} = -\, H_x M_s \int_0^\delta dz \int_0^\infty dr \int_{\rm semicirc} d\varphi \left\{ \left[r\cos\Theta(r) \frac{d\Theta(r)}{dr}\ \cos^2\varphi + \sin\Theta(r)\ \sin^2\varphi \right] \mathbf{e}_x \right.$$
$$\left. + \left[r\cos\Theta(r) \frac{d\Theta(r)}{dr} - \sin\Theta(r) \right] \sin\varphi\cos\varphi\ \mathbf{e}_y \right\} \tag{27}$$

where δ is the average thickness of the surface film layers where closure "cap" domains are present.

The limits in the φ-integration are either $-\frac{\pi}{2} < \varphi < +\frac{\pi}{2}$ or $+\frac{\pi}{2} < \varphi < \frac{3}{2}\pi$ depending on the topology of the defect (e.g. for the case sketched in Fig. 1, one has $-\frac{\pi}{2} < \varphi < +\frac{\pi}{2}$).

Now, taking into account that $\int d\varphi \sin\varphi\cos\varphi = \frac{1}{2}\sin^2\varphi$, by parity considerations it follows immediately that the contribution of the top film layers to the y-component of the external force is zero.

Using less trivial considerations, it turns out that also the contribution to the x-component vanishes.

This is proved as follows.

First, let us consider that $\int d\varphi \sin^2\varphi = \frac{1}{2}\varphi - \frac{1}{4}\sin(2\varphi)$ and $\int d\varphi\cos^2\varphi = \frac{1}{2}\varphi + \frac{1}{4}\sin(2\varphi)$.

After φ-integration, one then has

$$\mathbf{F}_{\rm ext}\Big|_{\rm top} = -H_x M_s\ \delta\ \frac{\pi}{2} \int_0^\infty dr \left[r\cos\Theta(r)\frac{d\Theta(r)}{dr} + \sin\Theta(r) \right] \mathbf{e}_x \tag{28}$$

It is now useful to define the two primitives $\mathcal{I}_s(r)$ and $\mathcal{I}_c(r)$

$$\mathcal{I}_s(r) = \int dr \sin\Theta(r) \tag{29}$$

$$\mathcal{I}_c(r) = \int dr\ r\cos\Theta(r)\frac{d\Theta(r)}{dr} = r\sin\Theta(r) - \mathcal{I}_s(r) \tag{30}$$

where the relationship between $\mathcal{I}_c(r)$ and $\mathcal{I}_s(r)$ was obtained integrating by parts.

Taking for $\Theta(r)$ the explicit form (7), and performing the r-integration, the primitive $\mathcal{I}_s(r)$ can be expressed as

$$\mathcal{I}_s(r) = \int dr \sin\Big[\frac{\pi}{2} + p\frac{\pi}{2}\tanh\Big(\frac{r-R}{\Delta}\Big)\Big] = \int dr\cos\Big[\ p\frac{\pi}{2}\tanh\Big(\frac{r-R}{\Delta}\Big)\Big]$$
$$= \Big\{ - Si\Big[\frac{\pi}{2} - \frac{\pi}{2}\tanh\Big(\frac{r-R}{\Delta}\Big)\Big] + Si\Big[\frac{\pi}{2} + \frac{\pi}{2}\tanh\Big(\frac{r-R}{\Delta}\Big)\Big]\Big\}\ \frac{\Delta}{2} \tag{31}$$

where $Si(x) = \int_0^x \frac{\sin t}{t} dt$ denotes the Sine Integral function. Note that the result in Eq. 31 holds irrespective of $p = \pm 1$. Namely, $\mathcal{I}_s(r)$ does not depend on the z-polarization in the domains.

Moreover, in the approximation of a domain wall width, Δ, much smaller than the stripe width, $2R$, the function $\sin\Theta(r)$ takes the form of a well localized and symmetric peak, centered at $r = R$. Therefore, the integrand in Eq. 28 is unaffected by the stripe domain structure: i.e., the r-integration in Eq. 28 can safely be limited between 0 and $2R$.

Now, we observe that in the approximation $\Delta \ll 2R$, of a domain wall width much smaller than the stripe width, one has

$$\big[\mathcal{I}_c(2R) - \mathcal{I}_c(0)\big] \approx -\big[\mathcal{I}_s(2R) - \mathcal{I}_s(0)\big] \tag{32}$$

and

$$\big[\mathcal{I}_s(2R) - \mathcal{I}_s(0)\big] \approx \; Si(\pi)\;\Delta = 1.852\;\Delta \tag{33}$$

One therefore obtains

$$\int_0^{2R} dr \Big[\sin\Theta(r) + r\cos\Theta(r)\frac{d\Theta(r)}{dr}] = \Big[\mathcal{I}_s(2R) - \mathcal{I}_s(0)\Big] + \Big[\mathcal{I}_c(2R) - \mathcal{I}_c(0)\Big] = 0 \tag{34}$$

and

$$\int_0^{2R} dr \Big[\sin\Theta(r) - r\cos\Theta(r)\frac{d\Theta(r)}{dr}] == \Big[\mathcal{I}_s(2R) - \mathcal{I}_s(0)\Big] - \Big[\mathcal{I}_c(2R) - \mathcal{I}_c(0)\Big] \approx 2 \times 1.852\;\Delta \tag{35}$$

From Eq. 34 it follows that the contribution of the top film layer to the x-component of the external force vanishes. Q.E.D.

In conclusion, we have found that, in the approximation $\Delta \ll 2R$ and irrespective of $p = \pm 1$, *the contribution from the top film layers to the external force is vanishing*

$$\mathbf{F}_{\rm ext}\Big|_{\rm top} = 0. \tag{36}$$

- *Bottom layers:* $\gamma = \pi$.

  This is another simple case, where $\Phi = \phi + \pi$. Therefore one has

$$\begin{aligned}\mathbf{F}_{\rm ext}\Big|_{\rm bottom} &= +\,H_x M_s \int_{t-\delta}^{t} dz \int_0^\infty dr \int d\varphi \\ &\times \Bigg\{ \Big[\; r\cos\Theta(r)\frac{d\Theta(r)}{dr}\;\; \cos^2\varphi + \sin\Theta(r)\;\; \sin^2\varphi\Big]\mathbf{e}_x \\ &\quad + \Big[r\cos\Theta(r)\frac{d\Theta(r)}{dr} - \sin\Theta(r)\Big]\sin\varphi\cos\varphi\;\mathbf{e}_y\Bigg\}\end{aligned} \tag{37}$$

  Using the same symmetry considerations as for the case of the top surface, one therefore obtains that *the contribution from the bottom film layers to the external force is vanishing*

$$\mathbf{F}_{\rm ext}\Big|_{\rm bottom} = 0 \tag{38}$$

- *Central layers:* $|\gamma| = \frac{\pi}{2}$.

  – (a) Head-to-head configuration. In this case, the φ-integration limits are $-\frac{\pi}{2} < \varphi < \frac{\pi}{2}$ and, taking into account the change of helicity at the vertex of the dislocation, (i.e., $\gamma = +\frac{\pi}{2}$ for $-\frac{\pi}{2} < \varphi < 0$ and $\gamma = -\frac{\pi}{2}$ for $0 < \varphi < \frac{\pi}{2}$), $\mathbf{F}_{\rm ext}$ can be expressed as

$$\mathbf{F}_{\rm ext}\Big|_{\rm central,head-to-head} = -H_x M_s \int_\delta^{t-\delta} dz \int_0^\infty dr$$

$$
\begin{aligned}
&\times\Bigg\{\int_{-\frac{\pi}{2}}^{0} d\varphi\Big[\sin\Theta(r) - r\cos\Theta(r)\frac{d\Theta(r)}{dr}\Big]\sin\varphi\cos\varphi\ \mathbf{e}_x \\
&-\int_{0}^{\frac{\pi}{2}} d\varphi\Big[\sin\Theta(r) - r\cos\Theta(r)\frac{d\Theta(r)}{dr}\Big]\sin\varphi\cos\varphi\ \mathbf{e}_x \\
&-\int_{-\frac{\pi}{2}}^{0} d\varphi\Big[\sin\Theta(r)\cos^2\varphi + r\cos\Theta(r)\frac{d\Theta(r)}{dr}\sin^2\varphi\Big]\mathbf{e}_y \\
&+\int_{0}^{\frac{\pi}{2}} d\varphi\Big[\sin\Theta(r)\cos^2\varphi + r\cos\Theta(r)\frac{d\Theta(r)}{dr}\sin^2\varphi\Big]\mathbf{e}_y\Bigg\}
\end{aligned}
\tag{39}
$$

– (b) Tail-to-tail configuration. In this case, the φ-integration limits are $\frac{\pi}{2} < \varphi < \frac{3}{2}\pi$ and, taking into account the change of helicity at the vertex of the dislocation, (i.e., $\gamma = +\frac{\pi}{2}$ for $\frac{\pi}{2} < \varphi < \pi$ and $\gamma = -\frac{\pi}{2}$ for $\pi < \varphi < \frac{3}{2}\pi$), $\mathbf{F}_{\text{ext}}$ can be expressed as

$$
\begin{aligned}
&\mathbf{F}_{\text{ext}}\Big|_{\text{central,tail-to-tail}} = -H_x M_s \int_{\delta}^{t-\delta} dz \int_{0}^{\infty} dr \\
&\times\Bigg\{-\int_{\frac{\pi}{2}}^{\pi} d\varphi\Big[\sin\Theta(r) - r\cos\Theta(r)\frac{d\Theta(r)}{dr}\Big]\sin\varphi\cos\varphi\ \mathbf{e}_x \\
&+\int_{\pi}^{\frac{3}{2}\pi} d\varphi\Big[\sin\Theta(r) - r\cos\Theta(r)\frac{d\Theta(r)}{dr}\Big]\sin\varphi\cos\varphi\ \mathbf{e}_x \\
&+\int_{\frac{\pi}{2}}^{\pi} d\varphi\Big[\sin\Theta(r)\cos^2\varphi + r\cos\Theta(r)\frac{d\Theta(r)}{dr}\sin^2\varphi\Big]\mathbf{e}_y \\
&-\int_{\pi}^{\frac{3}{2}\pi} d\varphi\Big[\sin\Theta(r)\cos^2\varphi + r\cos\Theta(r)\frac{d\Theta(r)}{dr}\sin^2\varphi\Big]\mathbf{e}_y\Bigg\}
\end{aligned}
\tag{40}
$$

On the basis of parity considerations about the φ-integration, one immediately finds that the Cartesian y-component of the external force identically vanishes, for both cases of a head-to-head and a tail-to-tail magnetization configuration.

Summarizing, the contribution from the central film layers to the external force can be written

$$
\mathbf{F}_{\text{ext}}\Big|_{\text{central}} = \begin{cases} +H_x M_s(t-2\delta)\displaystyle\int_0^{\infty} dr\Big[\sin\Theta(r) - r\cos\Theta(r)\frac{d\Theta(r)}{dr}\Big]\mathbf{e}_x & \text{(head} - \text{to} - \text{head)} \\ -H_x M_s(t-2\delta)\displaystyle\int_0^{\infty} dr\Big[\sin\Theta(r) - r\cos\Theta(r)\frac{d\Theta(r)}{dr}\Big]\mathbf{e}_x & \text{(tail} - \text{to} - \text{tail)} \end{cases}
\tag{41}
$$

Now, taking into account that, in the approximation of a domain wall width much smaller than the stripe width ($\Delta \ll 2R$), the function $\Theta(r)$ takes the form of a well localized and symmetric peak, centered at $r = R$, the integration range in Eq. 41 can safely be limited between 0 and $2R$. Taking Eq. 35 into account, one finally obtains

$$
\mathbf{F}_{\text{ext}}\Big|_{\text{central}} = \begin{cases} +(t-2\delta)\ (2\times 1.852\ \Delta)\ M_s H_x \mathbf{e}_x & \text{(head} - \text{to} - \text{head)} \\ -(t-2\delta)\ (2\times 1.852\ \Delta)\ M_s H_x \mathbf{e}_x & \text{(tail} - \text{to} - \text{tail)} \end{cases}
\tag{42}
$$

Namely, *the contribution from a central film region, with thickness $t' = (t-2\delta)$, to the external force is nonzero.* Finally, we note that the sign of the external force depends on the in-plane magnetization configuration, whether head-to-head or tail-to-tail, in the transition region between two opposite domains. In general, the force does not depend on the sign of the z-polarization parameter ($p = \pm 1$), see Eq. 42.

VI. APPENDIX B: EXPLICIT CALCULATION OF THE EXTERNAL FORCE FOR THREE DIFFERENT MAGNETIZATION CONFIGURATIONS OF THE CENTRAL FILM LAYER

In this section, we make a comparison between the contributions to the external force calculated for three different spin configurations of the central spin layer, as schematically depicted in Fig. 6. Owing to its symmetry, the idealized

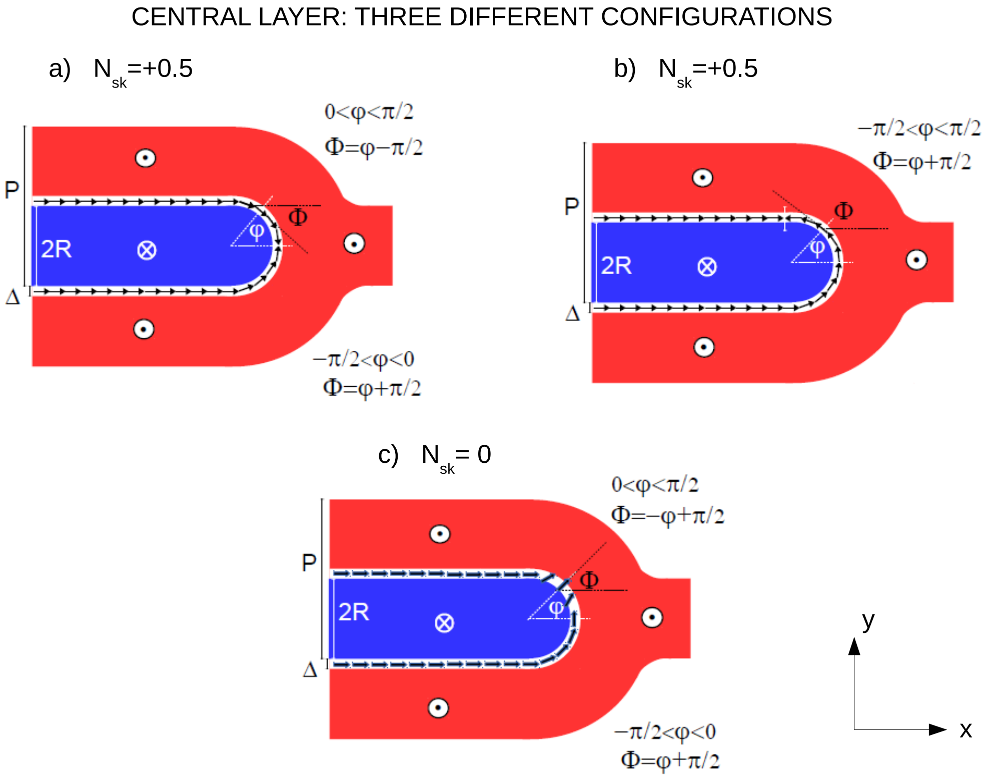

FIG. 6. **Three possible magnetization configurations for a magnetic edge dislocation in the central film plane.** Schematic view of three ideal possible configurations of the magnetization for a magnetic edge dislocation in the central film layer. The configurations have all the same $\Theta(r) = \frac{\pi}{2}\left[1 - \tanh\left(\frac{r-R}{\Delta}\right)\right]$ (i.e., $p = -1$) and three different $\Phi(\varphi) = m\varphi + \gamma$, where $m = \pm 1$ is the vorticity and γ is the helicity angle. We consider, respectively: (a) constant vorticity ($m = 1$) and a change of helicity ($\gamma = \pm\frac{\pi}{2}$) for $\varphi = 0$, leading to a topological number $N_{sk} = \frac{1}{2}$; (b) constant vorticity ($m = 1$) and constant helicity ($\gamma = \frac{\pi}{2}$), leading to $N_{sk} = \frac{1}{2}$; (c) a change of vorticity ($m = \pm 1$) for $\varphi = 0$ and constant helicity ($\gamma = \frac{\pi}{2}$), leading to $N_{sk} = 0$.

head-to-head configuration in Fig. 6a is expected to be realized at remanence, or for extremely small values of an in-plane dc magnetic field applied along the stripes axis. Whereas, the other configurations are likely to occur when the application of the external magnetic field produces deviations from the above idealized symmetric configuration.

We start from the general expression of the external force in Cartesian coordinates (14)

$$\begin{aligned}\mathbf{F}_{\rm ext} = & - H_x M_s \int_0^t dz \int_0^\infty r dr \int_{-\frac{\pi}{2}}^{+\frac{\pi}{2}} d\varphi \, \nabla\Big[\sin\Theta(r)\cos\Phi(\varphi)\Big] = -H_x M_s \int_0^t dz \int_0^\infty dr \int_{-\frac{\pi}{2}}^{+\frac{\pi}{2}} d\varphi \\ & \times\Bigg\{ \Bigg[r\cos\Theta(r)\frac{d\Theta(r)}{dr} \cos\Phi(\varphi)\cos\varphi + \sin\Theta(r) \sin\Phi(\varphi)\sin\varphi\frac{d\Phi(\varphi)}{d\varphi}\Bigg]\mathbf{e}_x \\ & + \Bigg[r\cos\Theta(r)\frac{d\Theta(r)}{dr} \cos\Phi(\varphi)\sin\varphi - \sin\Theta(r) \sin\Phi(\varphi)\cos\varphi\frac{d\Phi(\varphi)}{d\varphi}\Bigg]\mathbf{e}_y\Bigg\}\end{aligned} \tag{43}$$

where the φ-integration is extended to the semicircumference. Now we observe that, for all configurations in Fig. 6, one can write

$$\begin{aligned}\cos\Phi(\varphi) &= \cos(m\varphi+\gamma) = \cos\left(m\varphi + c\frac{\pi}{2}\right) = -\sin(m\varphi)\sin\left(c\frac{\pi}{2}\right) = -mc\sin\varphi \\ \sin\Phi(\varphi) &= \sin(m\varphi+\gamma) = \sin\left(n\varphi + c\frac{\pi}{2}\right) = +\cos(m\varphi)\sin\left(c\frac{\pi}{2}\right) = c\cos\varphi\end{aligned} \tag{44}$$

where $\gamma = c\frac{\pi}{2}$ and the parameter c is associated with a counterclockwise ($c = +1$) or clockwise ($c = -1$) rotation of the in-plane magnetization vector in the semicircular transition region between two opposite domains.

Taking into account that $\frac{d\Phi(\varphi)}{d\varphi} = m$, after some simple algebra one therefore obtains

$$\begin{aligned} \mathbf{F}_{\text{ext}} = &- H_x M_s \int_0^t dz \int_0^\infty dr \int_{-\frac{\pi}{2}}^{+\frac{\pi}{2}} d\varphi \\ &\times \Big\{ \Big[-r\cos\Theta(r)\frac{d\Theta(r)}{dr} + \sin\Theta(r) \Big] \, (\sin\varphi\cos\varphi) \, (mc) \, \mathbf{e}_x \\ &+ \Big[-r\cos\Theta(r)\frac{d\Theta(r)}{dr} \, \sin^2\varphi - \sin\Theta(r) \, \cos^2\varphi \Big] \, (mc) \, \mathbf{e}_y \Big\} \end{aligned} \tag{45}$$

- *(a) Central layer: constant vorticity ($m = +1$) and change of helicity ($c = \pm 1$) for $\varphi = 0$.*

  The first configuration in Fig. 6a is characterized by constant vorticity ($m = +1$) and by a change of sign in the helicity ($\gamma = \pm\frac{\pi}{2}$, or equivalently $c = \pm 1$) occurring just at the endpoint of the stripe domain (i.e., for $\varphi = 0$). The associated topological number is $N_{sk} = \frac{1}{2}$.

  For this head-to-head configuration, one has $mc = +1$ for $-\frac{\pi}{2} < \varphi < 0$ and $mc = -1$ for $0 < \varphi < +\frac{\pi}{2}$. The external force has already been explicitly calculated in the previous Appendix A and turns out to be directed along the stripes axis, $\mathbf{e}_x$, while the y component vanishes (see Eq. 39)

$$\mathbf{F}_{\text{ext}}\Big|_{\text{central}} = +(t - 2\delta) \, (2 \times 1.852 \, \Delta) \, M_s H_x \mathbf{e}_x \quad \text{(Fig. 6a)} \tag{46}$$

  where we remind (see previous Appendix A) that 2δ is the total thickness of the two surface film layers where closure "cap" domains are present, and the factor $(2 \times 1.852\Delta)$ is the result of the r-integration ($\Delta << 2R$ is the width of the transition region between two domains with opposite z component of the magnetization).

- *(b) Central layer: constant vorticity ($m = +1$) and constant helicity ($c = +1$) along the whole semicircumference.*

  The configuration in Fig. 6b is characterized by a constant vorticity ($m = +1$) and a constant helicity $\gamma = \frac{\pi}{2}$, or equivalently $c = +1$) in the whole semicircumference, $-\frac{\pi}{2} \le \varphi \le \frac{\pi}{2}$. In fact, a change of sign in the helicity ($\gamma = \pm\frac{\pi}{2}$, or equivalently $c = \pm 1$) is supposed to occur just for $\varphi = \frac{\pi}{2}$.

  The associated topological number is $N_{sk} = \frac{1}{2}$.

  Note that, in this case (b), one has $(mc) = 1$ in the whole range of integration $-\frac{\pi}{2} < \varphi < +\frac{\pi}{2}$. The explicit calculation of the external force leads to a vanishing result, for both the x and y components

$$\mathbf{F}_{\text{ext}}\Big|_{\text{central}} \equiv 0 \quad \text{(Fig. 6b)} \tag{47}$$

  The vanishing of the x component of the external force follows from the vanishing of the φ- integration, on the basis of simple considerations about the parity of the integrand. The vanishing of the y component of the external force follows from the vanishing of the r-integration

$$\int_0^\infty dr \Big[r\cos\Theta(r)\frac{d\Theta(r)}{dr} + \sin\Theta(r) \Big] = 0 \tag{48}$$

  The proof of the above result (48) proceeds as in the previous Appendix A, see Eqs. 28-36.

  Finally it can be shown that, in case the change of helicity occurred for an angle φ_0 different from $\frac{\pi}{2}$ (e.g. comprised in the range $0 < \varphi_0 < \frac{\pi}{2}$), both the x and y component of the external force would not vanish any more.

- *(c) Central layer: Change of vorticity ($m = \pm 1$) for $\varphi = 0$ and constant helicity ($c = +1$).*

  The configuration in Fig. 6c is characterized by a change of vorticity ($m = \pm 1$) occurring just at the endpoint of the stripe domain (i.e., for $\varphi = 0$) and by a constant helicity ($\gamma = \frac{\pi}{2}$, i.e. $c = +1$).

  Note that, in this case, the associated topological number vanishes, $N_{sk} = 0$.

  In this case (c), one has $mc = +1$ for $-\frac{\pi}{2} < \varphi < 0$ and $mc = -1$ for $0 < \varphi < +\frac{\pi}{2}$: namely, the product mc takes exactly the same values as in the case (a) of a head-to-head configuration calculated before. Therefore, the calculation of the external force, using Eq. 45, leads exactly to the same result for both configurations in

Fig. 6a and in Fig. 6c. Namely, the external force turns out to be directed along the stripes axis, $\mathbf{e}_x$, while the y component vanishes

$$\mathbf{F}_{\rm ext}\Big|_{\rm central} = +(t-2\delta)\ (2\times 1.852\ \Delta)\ M_s H_x \mathbf{e}_x \quad \text{(Fig. 6c)} \tag{49}$$

In conclusion, the external force, exerted on a dislocation by a dc magnetic field applied along the stripes axis, was found to be the same for two idealized configurations characterized by a different topological number (N_{sk}), but sharing a common feature: the product of vorticity and helicity changes sign ($m\gamma = \pm\frac{\pi}{2}$) at the vertex of the dislocation, see Fig. 6a and 6c. Whereas, for the configuration in Fig. 6b, where $m\gamma$ remains constant in sign and value along the whole semicircumference, the external force vanishes. Therefore, the product of vorticity and helicity, $m\gamma$, should be regarded as a parameter more relevant than just the topological number, N_{sk}, as far as a finite external force is concerned.

* mariagloria.pini@isc.cnr.it
† bisero@fe.infn.it
[1] L. D. Landau and E. M. Lifshitz, Phys. Zeit. Sow. **8**, 153 (1935), [English translation: Ukr. J. Phys. **53**, 14-22 (2008)], `http://www.ujp.bitp.kiev.ua/files/journals/53/si/53SI06p.pdf`.
[2] T. L. Gilbert, IEEE Trans. Magn **6**, 3343 (2004), `http://dx.doi.org/10.1109/TMAG.2004.836740`.
[3] L. Berger, Phys. Rev. B **54**, 9353 (1996), `http://dx.doi.org/10.1103/PhysRevB.54.9353`.
[4] J. C. Slonczewski, J. Magn. Magn. Mater. **159**, L1 (1996), `http://dx.doi.org/10.1016/0304-8853(96)00062-5`.
[5] S. Zhang and Z. Li, Phys. Rev. Lett. **93**, 127204 (2004), `http://dx.doi.org/10.1103/PhysRevLett.93.127204`.
[6] A. Thiaville, Y. Nakatani, J. Miltat, and Y. Suzuki, Europhys. Lett. **69**, 990 (2005), `http://dx.doi.org/10.1209/epl/i2004-10452-6`.
[7] R. Allenspach and P. Eib, Physics **3**, 91 (2010), `http://dx.doi.org/10.1103/Physics.3.91`.
[8] A. A. Thiele, Phys. Rev. Lett. **30**, 230 (1973), `http://dx.doi.org/10.1103/PhysRevLett.30.230`.
[9] A. A. Thiele, J. Appl. Phys. **45**, 377 (1974), `http://dx.doi.org/10.1063/1.1662989`.
[10] J. C. Slonczewski, J. Magn. Magn. Mater. **12**, 108 (1979), `http://dx.doi.org/10.1016/0304-8853(79)90005-2`.
[11] A. P. Malozemoff and J. C. Slonczewski, *Magnetic Domain Walls in Bubble Materials* (Academic Press, New York, 1979).
[12] W. Koshibae and N. Nagaosa, New J. Phys. **18**, 045007 (2016), `http://dx.doi.org/10.1088/1367-2630/18/4/045007`.
[13] S. Tacchi, S. Fin, G. Carlotti, M. Madami, M. Barturen, M. Marangolo, M. Eddrief, D. Bisero, A. Rettori, and M. G. Pini, Phys. Rev. B **89**, 024411 (2014), `http://dx.doi.org/10.1103/PhysRevB.89.024411`.
[14] S. Fin, R. Tomasello, D. Bisero, M. Marangolo, M. Sacchi, H. Popescu, M. Eddrief, C. Hepburn, G. Finocchio, M. Carpentieri, et al., Phys. Rev. B **92**, 224411 (2015), `http://dx.doi.org/10.1103/PhysRevB.92.224411`.
[15] A. Hierro-Rodríguez, C. Quirós, A. Sorrentino, C. Blanco-Roldán, L. M. Álvarez-Prado, J. I. Martín, J. M. Alameda, E. Pereiro, M. Vélez, and S. Ferrer, Phys. Rev. B **95**, 014430 (2017), `http://dx.doi.org/10.1103/PhysRevB.95.014430`.
[16] A. Hierro-Rodriguez, C. Quirós, A. Sorrentino, R. Valcárcel, I. Estébanez, L. M. Álvarez-Prado, J. I. Martín, J. M. Alameda, E. Pereiro, M. Vélez, et al., Appl. Phys. Lett. **110**, 262402 (2017), `http://dx.doi.org/10.1063/1.4984898`.
[17] N. Nagaosa and Y. Tokura, Nature Nanotech. **8**, 899 (2013), `http://dx.doi.org/10.1038/nnano.2013.243`.
[18] X. Zhang, M. Ezawa, and Y. Zhou, Sci. Rep. **5**, 09400 (2011), `http://dx.doi.org/10.1038/srep09400`.
